# Systematic review: The relationship between gabapentinoids, etizolam, and drug related deaths in Scotland

**DOI:** 10.1371/journal.pone.0310655

**Published:** 2024-10-09

**Authors:** Beata Ciesluk, Dr. Greig Inglis, Adrian Parke, Lucy J. Troup

**Affiliations:** University of the West of Scotland, School of Education and Social Sciences, Paisley, Scotland, United Kingdom; Lorestan University, ISLAMIC REPUBLIC OF IRAN

## Abstract

In recent years Scotland has been experiencing a disproportionally high number of drug related deaths compared to other European countries, causing significant individual, societal and economic burden. A possible cause of this is the increase in average number of substances involved in Scottish drug related deaths, as well as the changing pattern of substances involved. Opioids, cocaine, and alcohol have been consistently involved in the culture of drug use in Scotland, however recently National Records Scotland have identified that designer benzodiazepines such as etizolam, and prescription drugs such as gabapentinoids are increasingly being detected in Scottish toxicology reports. A systematic literature review following PRISMA guidelines was conducted through searching PubMed and Google Scholar to identify peer-reviewed articles published in English between 2013 and 2023 that investigated Scottish population data on gabapentinoids and etizolam to establish their contribution to the rise in Scottish drug related deaths. 18 studies were included in the review. A high use prevalence of etizolam and gabapentinoids in Scotland has been identified, with both substance-related deaths showing recent increase, marked since 2015. This pattern is replicated in the Scottish prison system. There has also been a significant increase of gabapentinoids prescriptions in Scotland. Polydrug use was identified as the most common determinant of both etizolam and gabapentinoids related adverse effects and fatality in Scotland, especially concurrent opioid use. The results indicate the literature on individual characteristics of Scottish at-risk users of gabapentinoids and etizolam is limited, however the data shows both substances are being used by older cohort, with adverse effects seen more in older women.

## 1. Introduction

In March 2021, the Scottish Parliament unanimously voted the Scottish rate of drug-related deaths (DRDs) a public health emergency [1]. In 2021 Scotland recorded the second highest annual DRD rate since the records began, doubling the recorded number from 10 years ago [2]. In 2020, Scotland recorded the highest DRD rate per capita in the EU with 245 deaths per million population which was 3.7 higher than the UK’s whole DRD rate [3]. The extent of Scotland’s DRD crisis is further illustrated through the comparison to the US which has been battling DRDs for two decades. In 2020, US recorded DRD rate of 277 deaths per million population, comparable with the rates recorded in Scotland [1]. Despite a 21% decrease of DRDs, in 2022 the number of DRDs in Scotland is still disproportionally high when compared to other countries, leading to a significant individual, societal, and economical burden [2]. Therefore, there is an unprecedented need to understand this devastating trend in Scotland.

The cause of the Scottish DRDs rate is complex. The House of Commons Scottish Affairs Committee [4] proposed a non-exhaustive list of possible explanations including poverty, policy, prescribing and polydrug use. One distinct feature of the recent Scottish rise in DRDs is the changing profile of substances involved in the deaths of people who use drugs (PWUD) and the increase in numbers of substances involved in Scottish DRDs. In 2008, on average, only one substance was found in 86% of DRDs however, by 2020, three or more substances were found in 61% of DRDs [5, 6]. Whilst cocaine and alcohol, alongside opioids have greatly affected the polydrug use trends in Scotland [5], recently it is particularly the pattern of use of designer benzodiazepines (DBZDs) and gabapentinoids that have changed the most [5–7]. This change in drug use patterns could be in part an explanation for the recent increase in DRDs.

The aim of this review is to provide an overview of these changes in drug use patterns in respect of the rise in DRDs in Scotland. The focus of this review centres on the rise of polydrug use and role of prescription drugs and designer benzodiazepines on DRDs in Scotland.

### 1.1. Designer benzodiazepines

In recent years there has been a significant increase in the use of so called “designer benzodiazepines” [8]. Benzodiazepines are currently one of the most globally prescribed medications for variety of conditions [8]. They have a central nervous system (CNS) depressant effect, producing calming and anxiolytic effects [8]. DBZDs emerged in early 2000’s as “legal alternatives” to their prescribed and controlled counterparts [8]. Recently, the market for DBZDs has increased significantly and is continually adapting to evade regulatory constraints [8, 9] which in turn increased DRDs. In 2020, benzodiazepines contributed to 73% of all global DRDs with DBZDs accounting for 66% of these fatalities [10]. Benzodiazepine related deaths are usually attributed to multidrug toxicity producing adverse effects such as respiratory depression [11]. The risk of benzodiazepine related deaths is amplified by the surge of high potency counterfeit tablets which are mimicking prescribed benzodiazepines such as diazepam [12]. These tend to include active adulterants and cutting agents which have the potential to enhance their harmful adverse reactions [12]. The risks caused by DBZDs in Scotland have followed the global trends leading to changes in prescribing guidelines in Scotland. Since these changes, the illicit market of DBZDs in Scotland has risen [13] with reports showing they were involved in most of DRDs in 2019 [14]. It is therefore important to understand how DBZDs are related to DRDs in Scotland.

### 1.2. Etizolam

One DBZD, “etizolam” has been detected in most DBRD’s globally, with significant levels being found in Scottish benzodiazepine related deaths [10–14]. Etizolam is an anxiolytic originally developed in 1984 and has similar pharmacological profile to other benzodiazepines working on the same neuropharmacological mechanisms, but with greater affinity, producing elevated euphoric effects [15, 16]. Etizolam, as an anxiolytic is also more potent than other benzodiazepines (e.g. diazepam), with 1mg of etizolam corresponding to 5g of diazepam [17]. Notably, pre-clinical evidence shows that etizolam is less lethal than other benzodiazepines, requiring larger dosing to cause death [18]. Due to the limited evidence that the pharmacological properties of etizolam solely contribute to its lethality there has been an increase in research on its role in increased mortality. Possible explanations relating to DRD in Scotland include its widespread availability, polydrug use and counterfeit supply [19].

#### 1.2.1. Etizolam widespread availability

The popularity and availability of etizolam is exceeding other DBZDs. This can be considered a factor in its role in DRDs in Scotland. The latest WEDINOS [20] report found that etizolam was the most common substance detected in drug samples seized by drug services, criminal justice authorities and health care providers across the UK [20]. Mullin et al. [10] further illustrated its popularity through web platforms analysis. Etizolam dominated social media discussions with 59% of all drugs related posts involving etizolam, with 24.2% of these posts informing individuals on purchasing and selling etizolam [10]. Its increasing popularity could be due to etizolam euphoric effects and assumptions of its lower lethality. The introduction of the UK Psychoactive Substance Act (PSA) in 2016 [8] was meant to curb the proliferation of DBZDs by restrictions on web based and international sales [21]. Nevertheless, etizolam is still being widely manufactured and purchased. This may be attributed to novel marketing strategies allowing anonymous purchase on the dark web [22, 23] or residual supplies circulating among illicit supply chains [10].

#### 1.2.2. Etizolam counterfeit supply

Further to its widespread availability the contribution of etizolam to DRDs could be linked to the supply of illicitly manufactured pills. This has the potential to cause inaccurate dosing, unintentional ingestion, and be associated with polydrug use. etizolam is often produced to mimic the look of the drug to the substances they are being substituted for. In the UK, in 2022, etizolam was detected in 22.6% of all tested tablets labelled as diazepam, in which, 53% had etizolam as the sole component [24]. Manufactured etizolam pills also vary in dosages and potency with some found to contain 20 times the recommended therapeutic dose of 4mg/day [24]. Therefore, individuals may be unknowingly consuming large doses of etizolam, or unintentionally consuming etizolam alongside or instead of other benzodiazepines. This then in turn could be linked to unintentional overdose or adverse effects [25]. The co-consumption of different benzodiazepines is concerning given they have different acting windows and adverse drug-to-drug interactions [26].

#### 1.2.3. Etizolam and poly-drug use

Polydrug use appears to be especially popular in Scotland, it is therefore unsurprising that etizolam has often been found to be consumed with other psychoactive substances [25] typically consumed alongside opioids, as well gabapentinoids, cocaine and other DBZDs [25–27]. Reasons for concurrent use usually include enhancing the effects of other drugs or to aid negative withdrawal symptoms [28]. Research interrogating social media and internet resources showed that etizolam polydrug use is a common trend, with many discussions found to inform users on best etizolam combinations such as etizolam and opioids, Xanax, and alcohol [10]. Furthermore, etizolam use alongside these drugs was reported to aid insomnia and anxiety symptoms, end a trip from high potency drugs or used as a “downer” to manage drug cravings [10]. As the use of DBZDs alongside other psychoactive substances has a compounding effect on the CNS depression, causing adverse drug interactions [29] the culture of polydrug may be contributing to the etizolam related deaths in Scotland.

#### 1.2.4. Current research on etizolam

Etizolam is a prevalent and potentially harmful drug, especially if consumed alongside other substances [10, 28, 29]. Many reviews have however highlighted the limited clinical and pharmacological evidence of etizolams effects, with most current studies using secondary data obtained from post-mortem or prescribing case analysis [18, 25, 26, 28, 29]. These are limited, including underreporting of DRDs, limited data on individual differences, and limited evidence on the potential long-term effects of etizolam [18, 29]. Furthermore, to our knowledge no systematic review to date has assessed the Scottish literature on the role etizolam has in the rise in DRDs in Scotland. Determining the extent and quality of Scottish literature is crucial, as identifying limitations and gaps are needed to address and aid the development of targeted education, harm reduction strategies and regulations. This then has the potential to safeguard individuals using etizolam in Scotland and limit the increases in fatalities.

### 1.3. Gabapentinoids

Another class of drug that appears to be having a significant effect on DRDs in Scotland are gabapentinoids. Pregabalin and gabapentin are widely used for the treatment of neuropathic pain and epilepsy disorders, with pregabalin recently approved for the treatment of generalised anxiety disorder. Additionally, several off-label uses of gabapentinoids include treatment for chronic non-cancer pain, insomnia, migraines, phobias, bipolar disorder, depression, and alcohol withdrawal [30, 31]. Gabapentinoids selectively bind to voltage-gated calcium channels in the CNS neural tissues which decreases excitatory neurotransmitters by increasing the GABA levels [32, 33]. It is through this mechanism that gabapentinoids can cause their antinociceptive, anticonvulsant, anxiolytic and sleep regulating effects [34]. Gabapentinoids are now among the most globally prescribed medications [35]. In 2016, gabapentin was one of the most prescribed drugs in the US with 64 million dispensed prescriptions [36] whereas pregabalin was marked in the top drugs in invoice spending in 2016 with estimated sales of 4.4 billion, doubling the sales recorded in 2012 [36]. The UK has shown a similar trend, with around 900% increase in prescriptions between 2008 and 2018 [37], and a 67% increase in new gabapentinoids patients in 2017 [38].

#### 1.3.1. Gabapentinoids related harms

Despite their reputation as safe drugs, gabapentinoids can cause adverse reactions, from sedation, dizziness, intoxication [33] to severe adverse CNS reactions, fatal cardiac effects, muscle control impairments [39, 40] and misuse leading to its abuse potential [41, 42]. In the UK, 2.5% of the general population is misusing gabapentinoids [43]. This prevalence is higher for individuals with substance use disorder (SUD), with misuse prevalence of 68% for pregabalin, and 22% for gabapentin [30]. Lastly there has been a worldwide increase in gabapentinoids related deaths (GRDs) [41], with Scotland GRDs increasing from 2 to 367 deaths between 2008 to 2018, accounting for 31% of all DRDs that year [7]. Notably, only a limited number of deaths involve solely gabapentinoids, with most deaths also involving opioids or benzodiazepines [7]. Despite their limited sole contribution to DRDs, GRDs are increasing rapidly, which has initiated a debate in the field on the causes for this phenomenon. It is likely that the increase in prescribing is related to its abuse potential, polydrug use and could be contributing to the rise in GRDs [36, 41, 42, 44–46].

#### 1.3.2. Gabapentinoids widespread availability

In 2019, The UK government has reclassified gabapentinoids as class C drugs to restrict their use and in turn highlight their role in drug related harm [47]. Nevertheless, their prescribing continues to increase, and is seen to be contributing to related harms. Kalk et al. [48] examined deaths following gabapentinoids use in England between 2004–2022, finding 2322 deaths with either prescribed or illicitly sourced gabapentinoids reported to be present, indicating that the UK prescribing could be contributing to the increase in GRDs. The recent initiatives in the UK to limit prescribing of opioids and benzodiazepines are thought to be driving the increase in gabapentinoids prescribing, especially their off-label use [48]. In 2017, in the UK, off-label prescribing accounted for 50% of gabapentinoids prescriptions [38], with most used for chronic non-cancer pain, despite the limited evidence of their effectiveness for this condition [46]. This off-label prescribing has been associated with misuse, increased diversion, and illicit-market availability. Gabapentinoids have been reported to be mostly obtained from GP’s or from diverted supply from family and friends [43], with gabapentinoids misusers also often reporting possessing a long term off-label prescription prior to misusing the drug, and that the widespread availability of gabapentinoids in the community was a significant driver for their misuse [49, 50].

#### 1.3.3. Gabapentinoids and polydrug use

Although there are risks associated with gabapentinoids and their role in poly drug use related harms such as overdose, adverse respiratory and cardiac effects [41] they are still frequently prescribed. In the UK, gabapentinoids are often prescribed as a part of a complex medicine regimen that includes benzodiazepines, antidepressants and opioids [51]. Montastruc et al. [38] found that between 2007–2017, in the UK, the rate of patients with opioid or benzodiazepine co-prescription increased by 162.4% for gabapentin and 217.4% for pregabalin [38]. The increased use of concurrent gabapentinoids prescriptions could reflect the attempts of avoiding increase of opioid or benzodiazepine doses, following the initiatives to limit their prescribing [48]. Evidence suggests that the changing prescribing landscape could have influenced the increase in Scottish DRDs. Their availability as a prescription medication has contributed to drug related harm. Data from opioid dependant patients has shown that individuals with concurrent gabapentinoids prescriptions are 30 to 40% more likely to abuse gabapentinoids compared to those without gabapentinoids prescriptions [30]. Furthermore, Gomes et al. [45] found that concomitant gabapentin use was associated with 60% increase in opioid related deaths, and 49% increase in opioid related overdose. Data from pre-clinical research shows that when using gabapentinoids in conjunction with opioids can reduce the effectiveness of opioid blockers such as naloxone which can further increase the risks of overdose [52]. This growing body of evidence indicates that increasing gabapentinoids prescriptions could be contributing to the rise in DRDs.

#### 1.3.4. Current research on gabapentinoids

Gabapentinoid related research is limited, especially in the UK [38, 42, 46, 53]. Data, mostly comes from secondary analysis of English postmortem, prescribing or overdose reports [38, 42]. Notably, these reports often omit registering detected gabapentinoids in DRDs, especially if opioids were involved, assigning the liability to opioid toxicity only [41, 48]. This may have contributed to the lack of awareness of gabapentinoids harms and in turn influenced increased prescribing and misuse in the UK. The motivation behind gabapentinoids use are also not clear. It has been shown that it is their heightened euphoric effects that is driving misuse [42], however, others have found that gabapentinoids are mostly misused in order to self-medicate symptoms of chronic pain [44]. Gabapentinoids have also been shown to potentiate the “high” and therapeutic effects of opioids and benzodiazepines, making them appealing for abuse among PWUD [53–55]. There is also conflicting data on the individual differences in gabapentinoid abusers. Some research indicates highest prevalence in young males [45, 56], with others finding a more diverse sample especially among PWUD, consisting of older individuals [57], and females [35–58]. Although there are various explanations for gabapentinoid misuse, it is the undervaluation of the risk of harm that seems to be driving their misuse and prescriptions patterns the most [40, 59]. Recently, UK prescribers have prompted for more research to establish whether gabapentinoid harms are surpassing their effectiveness [36, 59]. To our knowledge no systematic review to date has assessed the Scottish literature on gabapentinoids and their contribution to the rise in DRDs. An understanding of the role of gabapentinoids in DRD would benefit prescribers and policy makers to allow informed decisions on prescribing of gabapentinoids in Scotland.

### 1.4. Summary and research objectives

Etizolam and gabapentinoids use has substantially increased in Scotland, potentially influencing the rise in DRDs. This trend could be attributed to the recent changes in prescribing landscape, with the initiative to limit prescribing of opioids and benzodiazepines in the UK [13, 14, 48]. This could have prompted PWUD to seek other sedative-like substances such as DBZDs and gabapentinoids, with both being widely and easily acquired from black market supply [10, 49]. Gabapentinoid prescriptions have recently increased, amplifying their availability, misuse, and diversion [37, 38, 48]. Although gabapentinoids and etizolam are unlikely to cause death on their own they are still found to be involved in a substantial number of DRDs. There are many ways in which these drugs could be contributing to the rise in DRDs, with both substances being assumed as safe, and often concurrently used alongside each other and or opioids causing severe adverse reactions [29, 41]. It is therefore necessary to further understand this changing drug use patterns, especially in Scotland where the DRDs continue to grow.

Therefore, the present systematic review key objectives are to summarise the Scottish literature on gabapentinoids and etizolam and establish to what extent these substances are contributing to the rise in Scottish DRDs. The overarching aim is to provide a synthesised summary of the literature to inform policy makers and treatment providers of the risk from these drugs in relation to their role in DRDs.

## 2. Methods

### 2.1. Search strategy

A systematic literature review was performed on 18/10/2023 using the Preferred Reporting System Items for Systematic Reviews and Meta Analysis (PRISMA) checklist (Table 1 in S1 Table) to retrieve studies from Medline (Pub Med) and Google Scholar on the following two searches: “gabapentinoids”, “Scotland”, “Drug Related Deaths” and “benzodiazepines” “etizolam” “Scotland” “Drug Related Deaths”. Studies describing data about the topics of interest observed were considered for inclusion if they were reported in English. The exclusion criteria were studies that had not been peer reviewed and studies which did not include Scottish data or were not specifically related to a Scottish population, as well studies which did not investigate the drugs under review (gabapentinoids, etizolam). The search was spanned for articles published between 2013 to 2023. This review only included studies from 2013 onwards because this year followed the identification of increase in gabapentinoids and etizolam prevalence in Scotland [7, 14].

**Table 1:** Summary of general characteristics and results of eligible studies.

Author	Drug Type	Objective	Sample	Methodology	Key Findings
Arab et al (2021) [63]	etizolam	Investigation of polysedative use and the underlying cardiovascular pathologies in Scottish DRDs.	436 postmortem reports from NHS Fife region, Scotland.	Retrospective-cross sectional analysis of post-mortem reports between 2009–2019. Analysed data consisted of drug related deaths (DRDs) identified via international classification data (ICD-10). Socio-demographics and cardiovascular diseases (CVD) pathologies were extracted. Frequencies, descriptive statistics, and multiple regressions were conducted using SPSSv.26 to investigate predictive relationship between drug classes, sociodemographic and CVD severity.	Out of 436 cases, analysis revealed that benzodiazepines (alprazolam, diazepam, etizolam) were identified in 34.4% (n = 150) of cases. The presence of designer benzodiazepines (DBZD) and prescribed benzodiazepines (alprazolam, diazepam, etizolam) was found predictive of heightened CVD score. Analysis of R2 and effect sizes indicated notable impact of total CVD score, atheroma, and fibrosis pathologies, (later two not significant). Polydrug use was detected: 76.8% opioid co-use, 14% stimulant co-use, 27.1% alcohol co-use, 22% cannabinoid co-use, 22% anticonvulsants co-use.
Baird et al (2014) [64]	gabapentinoids (pregabalin and gabapentin)	Investigation of gabapentinoids abuse among patients with substance misuse problems in Scotland.	129 participants from six substance use clinics, Lothian Scotland.	Quantitative cross-sectional in person surveys on participants attending clinics between November 2011 and January 2012. Survey adapted from a preliminary study conducted on a single site over shorter period. Prescribed/non prescribed drug use was assessed. Free-text information was acquired to find motives for gabapentinoids use. Descriptive statistics were conducted on data using Excel 2007.	1.5% of sample reported pregabalin prescription, 7% reported gabapentin prescription, all prescription were for chronic conditions. 22% of sample reported non-prescribed gabapentinoids use (3% pregabalin, 19% gabapentin). All these participants reported current methadone prescription for opioid maintenance treatment. The motives for gabapentinoids misuse were mostly associated with enhancing methadone effects.
Bibi et al (2015) [65]	etizolam	Investigation of possible application of differential scanning calorimetry (DSC) as a fast and efficient tool for identification of contained drugs in Scotland.	16 cases of Diazepine tablets labelled A-P.	Analysis of the visual appearance, physical attributes, drug type/quantity and thermal properties was conducted on diazepine tablets obtained from police Scotland. Raw data was processed using Principal Component Analysis using Unscrambler X software. Statistical grouping of different cases using thermograms was obtained. HPLC analysis was used to analyse drug quantity. Differential scanning calorimetry (DSC) was used to analyse thermal properties.	HPLC analysis indicated that 75% of the ‘diazepam’ containing cases contained diazepam, but less than half of these contained the recognised amount of 10mg, with two cases (J/K) containing solely etizolam. Etizolam containing tablets (J/K) had markings of “MA/D10”. In cases containing diazepam (C) and phenazepam (B/E) these markings "MA/D10" were also observed.
Corkery et al (2023) [66]	etizolam, gabapentinoids	Investigation of drug-related poisoning deaths involving drowning in Scotland between 1996–2020.	162 cases of Scottish DRDs due to drowning.	Case series of DRDs due to drowning registered by NRS between 01/03/2013-21/12/2023 and 1996–2012. Information on death circumstances, socio-demographics, ICD-10 codes, and substances found in the body post-mortem was provided. Statistical analysis included frequencies, proportions, descriptive statistics, and Chi-square tests using Microsoft Excel v10.	49 cases included benzodiazepines (30.65). DBZD were most common type (57.1% of all benzodiazepines). Etizolam in 23 DBZD cases (46.9% of all benzodiazepine cases). Polydrug use was detected. Most common was alongside DZBD (including etizolam): opioid co-use (17.5%), alcohol (11.9%), antidepressants (11.9%). Annual variations in DRD rates detected with most increase in DRD between 2019–2020. DZBD (including etizolam) was the most significant factor in this increase. Gender differences were detected. 68.75% of the deceased were male, average age was 39.84, male descendants were younger than female descendants (38.12 vs 43.64). Female most common intent of death was intentional (50%), whereas males most common intent was accidental (44%).
Corkery et al (2022) [67]	etizolam, gabapentinoids (pregabalin and gabapentin)	Investigation of alprazolam related mortality cases in Scotland between 2013–2020.	366 cases of aloprozolam related deaths in Scotland.	Case series of data obtained from NRS covering deaths between 2013–2020. Individual differences and death circumstances were analysed descriptively. alprazolam and other benzodiazepine mortalities were described. Microsoft Excel 365 was used for all analysis.	From 366 cases, 77.9% were due to accidental poisoning. In these deaths, gabapentinoids were involved in 42.9% (pregabalin: 29.51%; gabapentin: 18.31%). “Other benzodiazepines” were involved in 67.21% (etizolam: 22.4%). Mean number of substances involved in one death reaching 5. 64.2% of cases involved “other benzodiazepines” and opiates. Regarding all registered deaths between 2013–2020, benzodiazepines solely implicated in 40 deaths, with any mention in 4245 deaths (etizolam sole implication: 53%, any mention 67.5%).
Corkery et al (2020) [68]	etizolam	Narrative on investigations into DRDs and how they aid the understanding of Novel Psychoactive Substances (NPS).	N/A	N/A	Found increase in DBZD related deaths between 2015–2016. 162% increase in etizolam and alprazolam deaths between 2017–2018. 94.53% of NPS deaths in Scotland between 2013–2018 included alprazolam, diazepam, etizolam.
Ghose et al (2022) [69]	gabapentinoids	Investigation of non-fatal overdoses (NFODs) experienced by individuals in a specialist community-based substance use disorder (SUD) service in Scotland.	N/A	Retrospective observational cross-sectional analysis of 555 patients who experienced NFOD between 2017–2019 using The Scottish Ambulance Service Data Base, Tayside Substance Misuse Service. Descriptive statistics assessed the demographics, Chi-quare tests assessed the relationship between NFOD/opioid use, Multivariate logistic regressions assessed risk NFOD risk factors, Odds Ratio and Confidence Intervals were by generalised linear models in R.	Gabapentinoids were involved in 61 NFOD cases (13.6%) with 60 cases due poly-drug use (methadone: 44 cases, heroin: 28 cases).
Kurdi (2021) [70]	gabapentinoids (pregabalin and gabapentin)	Investigation of opioids and gabapentinoids utilisation trends across the four United Kingdom countries and their correlation to related mortality.	N/A	Retrospective, observational, cross-sectional analysis using Prescription Cost Analysis datasets from four UK nations analysing trends occurring between 2010–2019. Opioids and gabapentinoids utilisation were measured using number of items dispensed/1,000 inhabitants and defined daily doses (DDDs)/1,000 inhabitant/day. Opioids and gabapentinoids related mortality data was extracted from the United Kingdom Office for National Statistics (2010–2018). Descriptive statistics, linear trend analysis, and line regression was used to analysed trends over time and obtain average annual changes in utilisation. Correlation between the substance utilisation and mortality was analysed through Pearson correlation.	There was an increase of 205.6% of total gabapentinoids dispended items/1000 inhabitants in the UK between 2010 to 2019. There was a significant annual increase of 93.5 dispensed items/1000 inhabitants (p<0.001) between 2010 to 2019. This increase was lower from 2016 to 2019, compared to 2010 to 2016, of 39.6 dispensed items/1000 inhabitants (p<0.001). Considering dispensed items/1000 inhabitants, pregabalin accounted for majority of gabapentinoids use in Scotland. There was an overall increase of 207% of total ggabapentinoids utilisation rate of DDDs/1000 inhabitants/day in UK between 2010 to 2019. Scotland had the second highest utilisation rate for pregabalin, and the highest increase overall. Gabapentin had the highest utilisation rate in Scotland, and the highest increase rate in par with Wales. Gabapentinoids related deaths increased 12-fold in Scotland over study period, with an annual increase rate of 8.7 deaths per million inhabitants, with highest increase observed after 2015.
Lowrie et al (2023) [71]	gabapentinoids (pregabalin and gabapentin)	Description of baseline findings from an ongoing pilot randomised control trial (RCT) in Glasgow (PHEONIX) with homeless individuals living in Glasgow.	128 homeless individuals from Glasgow with a recent NFOD (past 6-months).	Observational analysis of baseline characteristics from pilot RCT, recruitment occurred between May-September 2021. Data obtained from interviews/clinical/social records Descriptive outcomes were analysed using MINITAB statistical software (version 21).	128 participants had at least one NFOD in the last 6-months. Mean age was 42. Participants used a median of three different street drugs in addition to Opioid Substitute Therapy, diazepam and in one case diamorphine from the Heroin Assisted Treatment Unit. The mean (SD) number of overdoses in the past 6-months was 3.2. Study evaluated the patterns of use 20% of participants reported that they have bought and took street pregabalin and gabapentin.
Marland et al (2023) [72]	etizolam	Investigation of DBZD prevalence in Scottish prisons, different use methods, drug search practices and legislation changes.	495 DBZD samples from 11 Scottish prisons.	Observational cross-sectional analysis of DBZD samples seized between (2019–2022). Detection and analysis of textiles, papers, blotters, cards were conducted. etizolam quantitation was performed using gas chromatography–mass spectrometry (GC–MS). All Qualitative data was processed by R.	Between 2019–2020 DBZD detection increased, accounting for 20% of all samples detected. Etizolam found in 301 samples (60% of all DBZD). Combinations of etizolam and flubromazepam found in 57 samples (11% of all DBZD). Etizolam was most prevalent between 2020–2021. Total of 193 etizolam samples were quantified. etizolam samples had high variability (0.03–2.33mg). Across a card etizolam concentration ranged from 1.16–1.87mg\cm2, whole card had 312.5mg of etizolam (i.e. 306 tablets).
McAuley et al (2022) [6]	etizolam	A commentary on the changing roles and patterns of benzodiazepine usage and prevalence in Scotland.	N/A	N/A	Etizolam first detected in Scottish DRDs in 2012 (0.2% of DRDs, 1/581). Increase in detection since 2015 (6% of DRDs, 43/706), further increase in 2020 (60% of DRDs, 806/1339). In 2020 only 1% of etizolam related deaths were due to sole etizolam use.
McAuley et al (2015) [73]	etizolam	Investigation of DRDs involving Novel Psychoactive Substances (NPS) in Scotland	N/A	Observational cross-sectional analysis of NPS related deaths in 2012 recorded by the Scottish National Drug Related Death Database comparing Scottish NPS related deaths to NPS implicated deaths, investigating sociodemographic and death circumstances, data analysis was conducted in SPP through descriptive statistics and exploratory analysis of DBZD and NPS differences.	Analysis revealed that in 2012 majority of NPS-related deaths were DBZD deaths (24 out of 36) with phenazepam accounting for 23 deaths, and etizolam accounting for one death.
Parks et al (2015) [74]	etizolam, gabapentinoids (pregabalin)	Investigation of post-mortem cases involving the use of ethylphenidate in East and West Scotland.	19 cases of ethylphenidate related deaths in east/west of Scotland.	Retrospective case collection utilising in house database of unexpected/sudden/unexplained ethylphenidate deaths between July 2013-December 2014 in Scotland. Drug presence was confirmed using liquid chromatography-tandem mass spectrometry. Sociodemographic, death circumstances, pathologies, toxicology, adverse reactions and injection sites were presented.	Etizolam detected in 2 cases, pregabalin detected in 2 cases. Multidrug toxicity was the sole contributing factor to the cause of death in 10 cases.
Rahman et al 2021 [75]	gabapentinoids (gabapentin and pregabalin)	Investigation of the annual rate of patients newly prescribed gabapentinoids in four UK nations (England, NI, Scotland, and Wales) and investigation of the proportion of patients concomitantly prescribed opioids.	Cohort of 12 512 468 NHS patients in the UK registered at least 1 day between 1993–1997.	Retrospective cohort study utilised data from clinical practice research data link of electronic medical records from UK (11% in Scotland). To identify new gabapentinoids patients, participants were excluded if they had gabapentinoids prescription prior to cohort entry. The crude annual rates and 95% CI were estimated, poison regression models were conducted to estimate the rate ratio (RR) between 2007–2017, adjusted for gender/age. Secondary analysis estimated crude annual rates and CI for concurrent opioid prescriptions.	The annual rate of patients newly prescribed gabapentin in Scotland gradually increased for most of the study period and plateaued between 2015 to 2017. Between 2007 and 2017 gabapentin rates increased from 369 to 742 per 100 000 PY (RR: 2.25; 95% CI: 2.21–2.28). The rate of concomitant opioid prescription changed proportionally with gabapentin prescriptions in Scotland. The annual proportion of concomitant users ranged from 15–20% over the study period (2007–2017). During the study period a steady increase in annual rate of Scottish patients newly prescribed pregabalin was detected. Between 2007–2017 the rate of pregabalin increased from 95 to 418 per 100 000 PY (RR: 4.7; 95% CI:4.69–4.85). The rate of concomitant opioid prescription changed proportionally with new pregabalin prescriptions, with annual proportion of concomitant users ranging between 15–20%.
Schofield et al (2021) [76]	gabapentinoids	Investigation of strong opioid prescribing and risk factors associated with overdose in regional health authority in Scotland.	46 GP practices in NHS, Fife, Scotland.	Observational cross-sectional analysis using data from GP practices, practices identified patients with at least one strong opioid prescription in the past 6 months (alone or with gabapentinoids, benzodiazepines, z-drugs) and their characteristics (e.g. overdose risk factors). Descriptive statistics were used to analyse risk factors for strong opioid prescriptions, linear regression and logistic models were used to investigate relationship of strong opioids and the practice/patient level factors.	Out of 341 240 patients, 12.4% were prescribed opioids, with 1/3 of them receiving strong opioids. Strong opioids were frequently prescribed in combination with other medications. The combination of strong opioids and gabapentinoids occurred in 9.36% of cases. Strong opioids in combination with benzodiazepines, z-drugs, gabapentinoids occurred in 2.62% of cases.
Torrance et al (2020) [77]	gabapentinoids (gabapentin and pregabalin)	Investigation of gabapentinoids prescribing increase in Scotland, and its associated sociodemographic, co-prescriptions and mortality.	N/A	Observational cross-sectional analysis using data from the Information Service Division Scotland. Analysis of prescribing trends, sociodemographic and mortality trends using data from the Health Informatics Centre, Dundee. Gabapentinoids deaths were analysed using NRS and Tayside Drug Death Data base. Descriptive analyses were used to examine gabapentinoids prescribing trends between 2006–2016 across Scotland, NHS Tayside, and NHS Fife. Demographics, prevalence of gabapentinoids recurrent users and co-prescribing of opioids and benzodiazepines in 2016 was investigated. Multiple logistics regressions were used to examine the association of age, gender, and SIMD with gabapentinoids recurrent users of gabapentinoids with co-prescribing. All analysis were conducted using IMB SPSS and Open Epi.	Gabapentin prescriptions increased in all areas from 2006 to 2016; In Scotland prescription increased from 16481 to 65241, In Tayside from 16481 to 57472, In Fife from 20465 in 2010 to 65241 in 2016. In Tayside and Fife, in 2016, 29111 (3.7%) of NHS patients were prescribed gabapentinoids (73.2% recurrent users, mean age was 58.1, 62.8% females, 24.6% lived in deprivation). 60% of this sample received co-prescriptions (opioids 49.9%, benzodiazepines 26.8%). Benzodiazepine co-prescribing was higher in females (28.5% vs 24.2%; p<0.05). Co-prescribing was higher in older population, especially older females (63.5% vs 36.4%; p < 0.001). 1312 deaths were identified (4.5% of gabapentinoids prescriptions) with 54 (4.1%) of those deaths classed as drug related. Higher gabapentinoids related deaths increase in 2017 in Tayside compared to Scotland (gabapentin 23% vs 14%; pregabalin 33% vs 12%). In Tayside, gabapentinoids related deaths consisted of 39% of all DRDs in 2016 (77% had no presctiprion). Gabapentinoids deaths in Tayside, in 2016 consisted of mostly younger population (27.2 vs 40.2), males (82% vs 76%) and deprivation.
Tweed et al (2022) [78]	gabapentinoids (gabapentin and pregabalin)	Investigation of the increase in women DRDs in Scotland.	N/A	Mixed-method investigation into the causes of the increase in women DRDs in Scotland. Three methodological strands were included: comprehensive analysis of routine data, secondary analysis of surveys with individuals using needle exchange programmes, thematic analysis of interviews with professional stakeholders recruited from the Partnership for Action on Drugs in Scotland Harm Reduction Group, and a local alcohol and Drug Partnership, a secondary analysis of interviews with women who use drugs from another project (Older People with Drug Problem). Descriptive statistics were used to analyse the routine data, which was narratively described, and the qualitative data was coded using direct context analysis based on a produced thematic matrix.	Found increase in attendance to treatment initiation meeting for gabapentinoids misuse support for males and females (higher increase rate for males). NRS data analysis revealed that gabapentinoids related deaths were increasing for both males and females, however the spike was much higher for men. ONS data showed smaller gender gap, where prescription gabapentinoids were included in the deaths. Stakeholder interview analysis revealed that prescription drugs were identified as a potential risk factor in the observed increasing DRDs for women, with particular concern including substances such as gabapentinoids and benzodiazepines.
Van Amsterdam et al (2021) [79]	etizolam, gabapentinoids	Investigation of the increase in Opioid Related Deaths (ORD) observed in Scotland, with comparison to ORD in England.	14 studies.	A systematic literature review conducted according to PRISMA guidelines. In addition to primary search on PubMed and PsycINFO, non-peer-reviewed reports on nationwide statistical data were retrieved via Google and Google Scholar and analysed to quantify differences in ORD drivers in Scotland and England/Wales. The search was limited to articles published from 2010 to 2020. 348 studies were retrieved out of which 234 were processed. After applying exclusion/inclusion criteria 14 studies were included in the review.	Black market was found the most common source of etizolam/gabapentinoids in Scotland. NRS review data showed that in 2018 DBZD (mainly etizolam) were found implicated in 67% of all DRDs in Scotland, and in 78% in all ORDs in Scotland. In 2018 gabapentinoids were found implicated in 31% of all DRDs in Scotland, and in 36% of all ORDs in Scotland. Combination of heroin/gabapentinoids was very common with 23% implicated in DRDs in Scotland in 2016. In 2018 methadone/gabapentinoids combination found to be more popular in older population (34%) compared to younger population (22%).

### 2.2. Articles screening and data extraction

One researcher (B.C) was involved in the selection of appropriate studies, which was executed in two rounds. Firstly, the results retrieved from the two searches were exported into End Note X10 and merged, after which any duplicates were removed. The titles and abstracts of the remaining studies were screened against the eligibility criteria to identify potentially relevant studies. The full text of the relevant studies was then obtained and comprehensively reviewed to check their eligibility. The following data was then extracted from the eligible studies: authors, year of publication, aim, design, results, and conclusions.

### 2.3. Risk of bias

The Joanna Briggs Institute (JBI) Critical Appraisal Tools (CAT) checklists [60] were used to assess the risk of bias (ROB) of included studies. The JBI CAT checklists were used in this review as they were research-specific and have been recently revised to adhere to the expansion of research-synthesis [60]. Furthermore, as this review included a variety of methodological designs the JBI CAT were selected due to their vast application range [61]. CAT for observational, analytical cross-sectional studies, CAT for observational, case series, CAT for textual evidence, CAT for systematic reviews and CAT for observational, cohort studies were applied as appropriate.

Every item of the JBI CAT checklist was answered with either yes, no, unclear or N/A answers. The scoring system and cut off points to determine high, moderate, or low ROB was decided based on previous studies [62] and agreed by two authors (BC, LJT) prior to the commencement of critical appraisal as recommended by the JBI manual [60]. The studies were then independently apprised by two authors (BC, LJT), and any disagreements were discussed until a consensus for each score was reached.

Quality assessment score for each study was assigned based on the calculated percentage of affirmative responses to the total number of questions. If a criterion was considered not applicable, this point was deducted from the overall score. Studies with JBI score higher than 66% were at low risk of bias, scores between 33 to 66% were at moderate risk of bias and studies with JBI score less than 33% were deemed as high risk of bias [62]. However, this review did not exclude any studies due to the quality assessment to ensure all potential studies can be used to produce a comprehensive review of the current research.

## 3. Results

The results section is separated into an overview of the studies to be analysed including Table 1. which summarises the results according to PRISMA guidelines. Following from this risk of bias is assessed highlighting limitations on the studies included in the review. The remainder of the results section includes a discussion of the data in relation to the themes arising from the studies included in the review.

### 3.1. Search results

**Fig 1:**
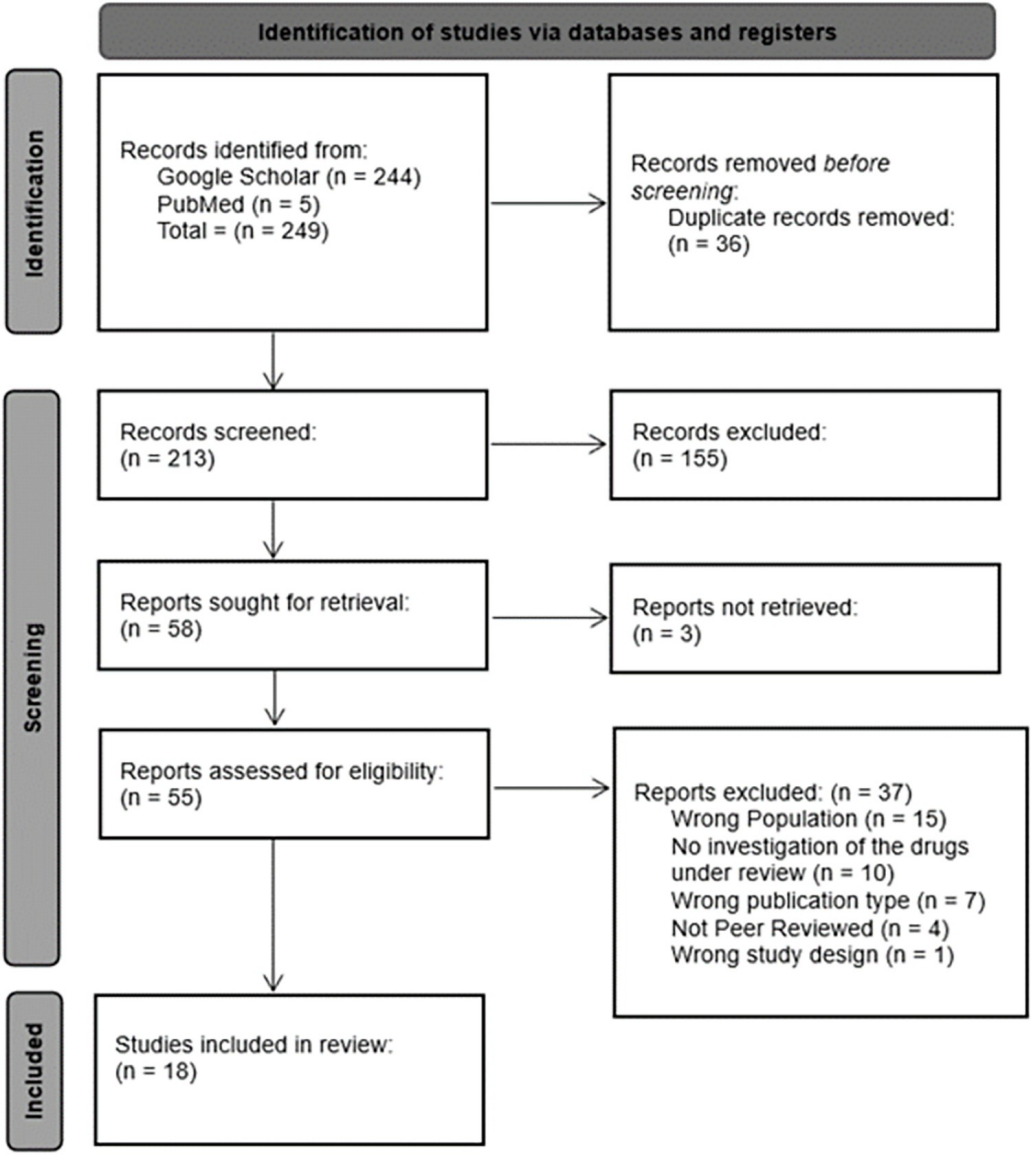
PRISMA flow diagram.

249 studies were retrieved through initial database search, of which 213 studies remained after duplicates were removed. The title and abstract screening were completed on the 213 studies. In the second round, full-text screening was applied to the selected 55 studies. Three studies were not included in the full-text screening due to the inability of full text-retrieval. After two rounds of screening, 18 papers were included in the review. Fig 1 shows the PRISMA diagram of the identification, screening, and inclusion of the reports.

### 3.2. Summary of studies

The articles selected for this review are summarised in Table 1, detailing the author, type of drug under investigation, methods, and main findings.

Studies consist of 14 observational designs including 10 analytical cross-sectional investigations [63–65, 69–73, 76, 77] three case series [66, 67, 74] and one cohort study [75], three reviews were also included; one systematic review [79], one narrative review [68] and one commentary [6], finally one mixed-method investigation was also included in the review [78].

11 studies were used to investigate gabapentinoids, of which six specified pregabalin and gabapentin, [66, 64, 70, 75, 77, 78], one study specified pregabalin only [74] and four did not specify the gabapentinoids [69, 71, 76, 79]. Nine studies were used to investigate etizolam [6, 63–68, 72–74].

### 3.3. Risk of bias

The calculated quality percentage and assigned ROB is represented in Table 2 for all but one study. Tweed et al. [78] had included a vast methodological design which did not fit the available JBI CAT checklists, thus after careful consideration decision was made to apprise the study narratively. Reviewers BC and LJT propose that the study has a rigorous methodology design and had been subjected to thorough peer-review system, therefore it has been agreed that the study was at low risk of bias. Considering the rest of the studies in this review, a further 11 studies were at low ROB, [6, 63, 65, 68–70, 72, 74–76, 79] whereas five studies were of Moderate ROB [66–67, 71, 73, 77] and one study was of high ROB [64]. The complete appraisal process for each study is represented in Table 2 in S1 Table.

**Table 2:** Quality score and risk of bias in the eligible studies.

Author/Year	JBI Critical Appraisal Tool	Quality Score	Risk of Bias
Arab et al (2021) [63]	Observational, analytical cross-sectional	75%	Low
Baird et al (2014) [64]	Observational, analytical cross-sectional	25%	High
Bibi et al (2015) [65]	Observational, analytical cross-sectional	75%	Low
Corkery et al (2022) [66]	Observational, case series	60%	Moderate
Corkery et al (2023) [67]	Observational, case series	60%	Moderate
Corkery et al (2020) [68]	Narrative Review	83%	Low
Ghose et al (2022) [69]	Observational, analytical cross-sectional	87.5%	Low
Kurdi (2021) [70]	Observational, analytical cross-sectional	87.5%	Low
Lowrie et al (2023) [71]	Observational, analytical cross-sectional	62.5%	Moderate
Marland et al (2023) [72]	Observational, analytical cross-sectional	75%	Low
McAuley et al (2022) [6]	Expert Opinion/Commentary	100%	Low
McAuley et al (2015) [73]	Observational, analytical cross-sectional	50%	Moderate
Parks et al (2015) [74]	Observational, case series	70%	Low
Rahman et al (2021) [75]	Observational, cohort study	90.9%	Low
Schofield et al (2021) [76]	Observational, analytical cross-sectional	75%	Low
Torrance et al (2020) [77]	Observational, analytical cross-sectional	63%	Moderate
Tweed et al (2022) [78]	N/A	N/A	Low
Van Amsterdam et al (2021) [79]	Systematic review	75%	Low

### 3.4. Analysis of results

As the included articles had a vast heterogeneity in designs and results it was out of scope for this review to conduct a statistical analysis of the data set, therefore a narrative review of the evidence was conducted to summarise the data from Scottish populations on etizolam and gabapentinoids exploring the following emerging themes: etizolam use prevalence, gabapentinoids use prevalence, determinants of adverse effects and demographics of at-risk users. Microsoft Excel was used to create certain graphs on data derived from articles, this was done by copying the data from tables and creating a graph for a visual representation.

### 3.5. Etizolam use prevalence

No studies were identified which investigated etizolam use patterns, therefore literature on fatal trends and seized samples was reviewed to establish etizolam prevalence in Scotland.

#### 3.5.1. Fatal trends

Data on etizolam fatal trends was derived from two observational analysis on Scottish DRDs [67, 73] and two reviews [6, 68]. One review investigated all benzodiazepines related deaths in Scotland [6], the other focused on Novel Psychoactive Substances (NPS) related deaths [68]. NPS are a broad term which describes compounds designed to mimic existing recreational drugs, including but not limited to DBDZ.

**Fig 2:**
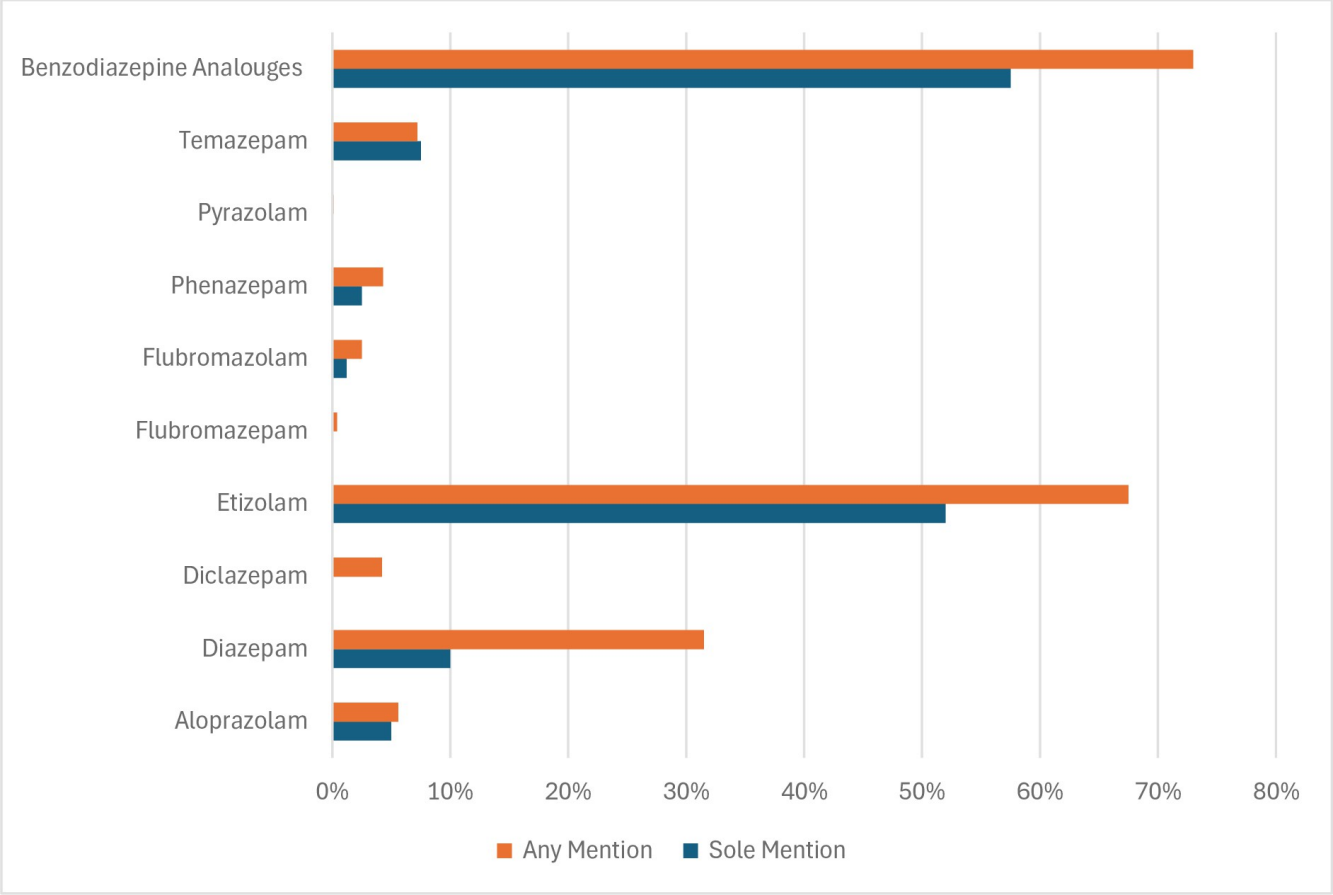
Percentage of Scottish cases where specific benzodiazepines were mentioned in the cause of death between 2013–2020. Source: Adapted from Corkey et al., (2022) original table. Licensed under CC BY 4.0.

Fig 2 shows prevalence of etizolam implications in Scottish deaths between 2013–2020, compared to other benzodiazepines.

Etizolam was the second most common benzodiazepine reported in the cause of death, with sole mentioning in 53% out of 40 sole benzodiazepines mentioning, and in 67.5% out of all 4256 cases of any mentioning of benzodiazepines. The highest proportion of any or sole mentioning of benzodiazepines involved “benzodiazepine Analogues” i.e. DBZD (Fig 2). As etizolam is a type of analogue, it’s prevalence rate in these deaths could be higher [67].

Two reviews exemplified timeline of etizolam prevalence in Scotland [6, 68]. Etizolam detection alongside other DBZD in NPS related deaths surged since 2015, and persisted to increase thereafter, being detected in 97.5% of all NPS related deaths by 2018. Etizolam was first detected in 2012 in 0.2% of 581 all DRDs, with a surge in 2015 representing 6% of 706 DRDs reaching 60% of 1339 DRDs in Scotland by 2020 [6, 68].

Investigation of DRDs involving NPS including DBZD corroborated this timeline, showing that etizolam was involved in only one NPS related death in 2012, compared to other DBZD being involved in 80% of these deaths [73].

#### 3.5.2. Seized samples

This review identified two analytical cross-section investigations which analysed etizolam quantitation in Scotland [65, 72].

An analysis of 16 cases of tablets labelled as “diazepam” seized by Police Scotland in 2015 revealed that diazepam was detected in 75% of these cases, with two cases containing solely etizolam tablets, and other containing large etizolam quantities, showing a growing circulation of etizolam supply at the time of the study [65].

**Fig 3:**
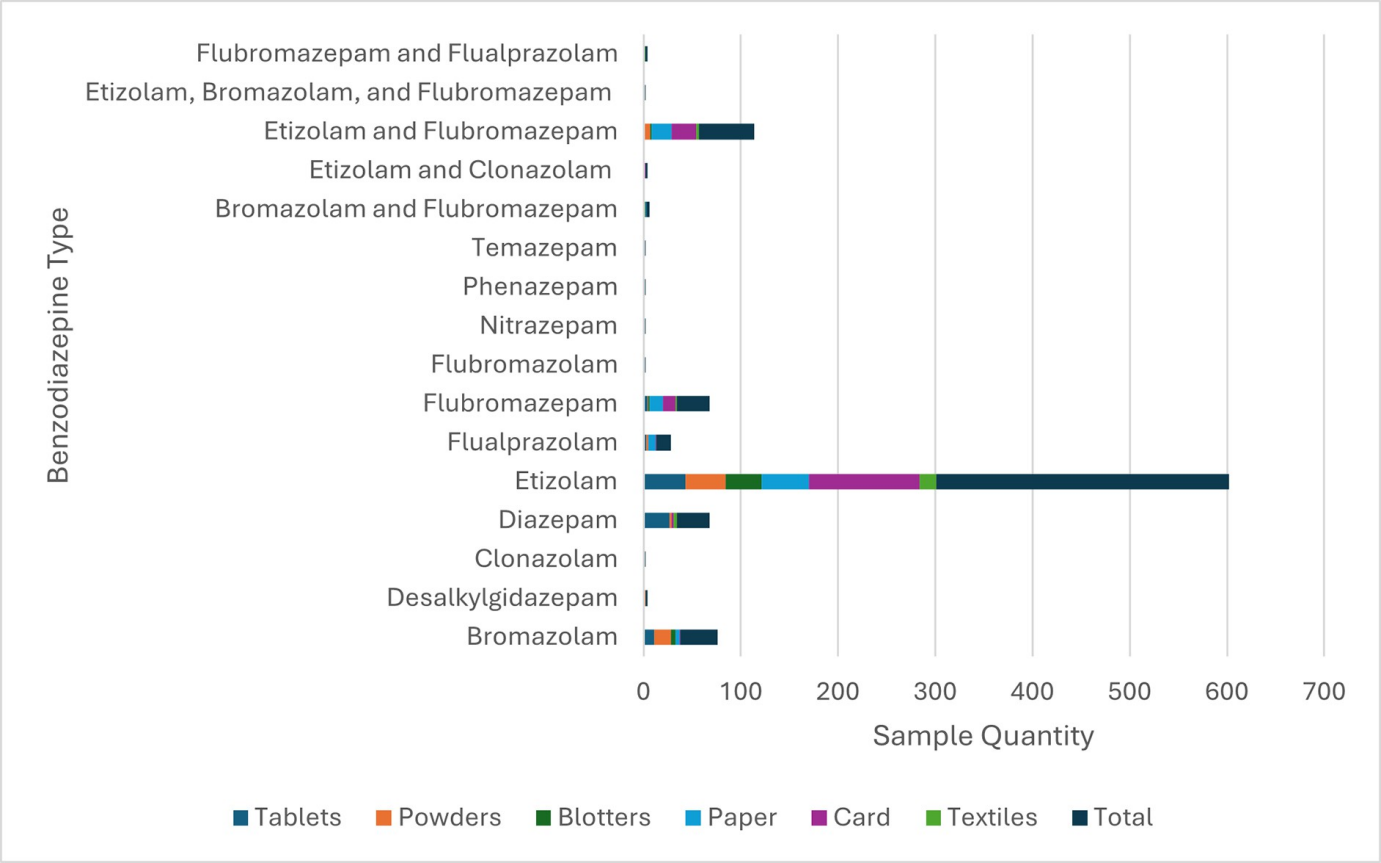
Sample types and quantity of benzodiazepines detected from Scottish prisons between Jan 2019 and Jan 2023. Source: Adapted from Marland et al (2023) original table. Licensed under CC BY 4.0.

Analysis of 3700 illicit drug samples seized in 11 Scottish prisons between 2019–2022 detected 495 DZBD, with etizolam accounting for 60% of these samples [72]. Fig 3 represents the quantity and type of all DZBD detected between 2019–2023, showing etizolam predominance in card and paper samples, and being the most prevalent DZBD across all sample types.

Etizolam was first detected in powder covered paper in 2019, with first detection of etizolam in infused greeting cards in 2020. Between January 2021 and December 2021 this was the most in common form of DZBD detection, showing expansion of etizolam smuggling techniques [72] and corroborating the recent increase in etizolam fatalities, with highest increase seen in 2020. Etizolam detection in Scottish prisons decreased since 2022, with a surge of novel DBZD entering northern prisons (e.g. bromazolam). However, there was a recent decrease in seized samples sent for testing which affected detection rates [72].

### 3.6. Gabapentinoids use prevalence

Scottish Literature on gabapentinoids use prevalence is limited, therefore data on prescription utilisation and fatal trends was reviewed to establish gabapentinoids prevalence in Scotland.

Two studies investigated the use of gabapentinoids in individuals with SUD, both showing that within SUD populations in Scotland, around 20% self-reported gabapentinoids misuse [64, 71] however this was found to be driven by gabapentin, especially for individuals with methadone dependence [64].

#### 3.6.1. Prescription utilization

**Fig 4:**
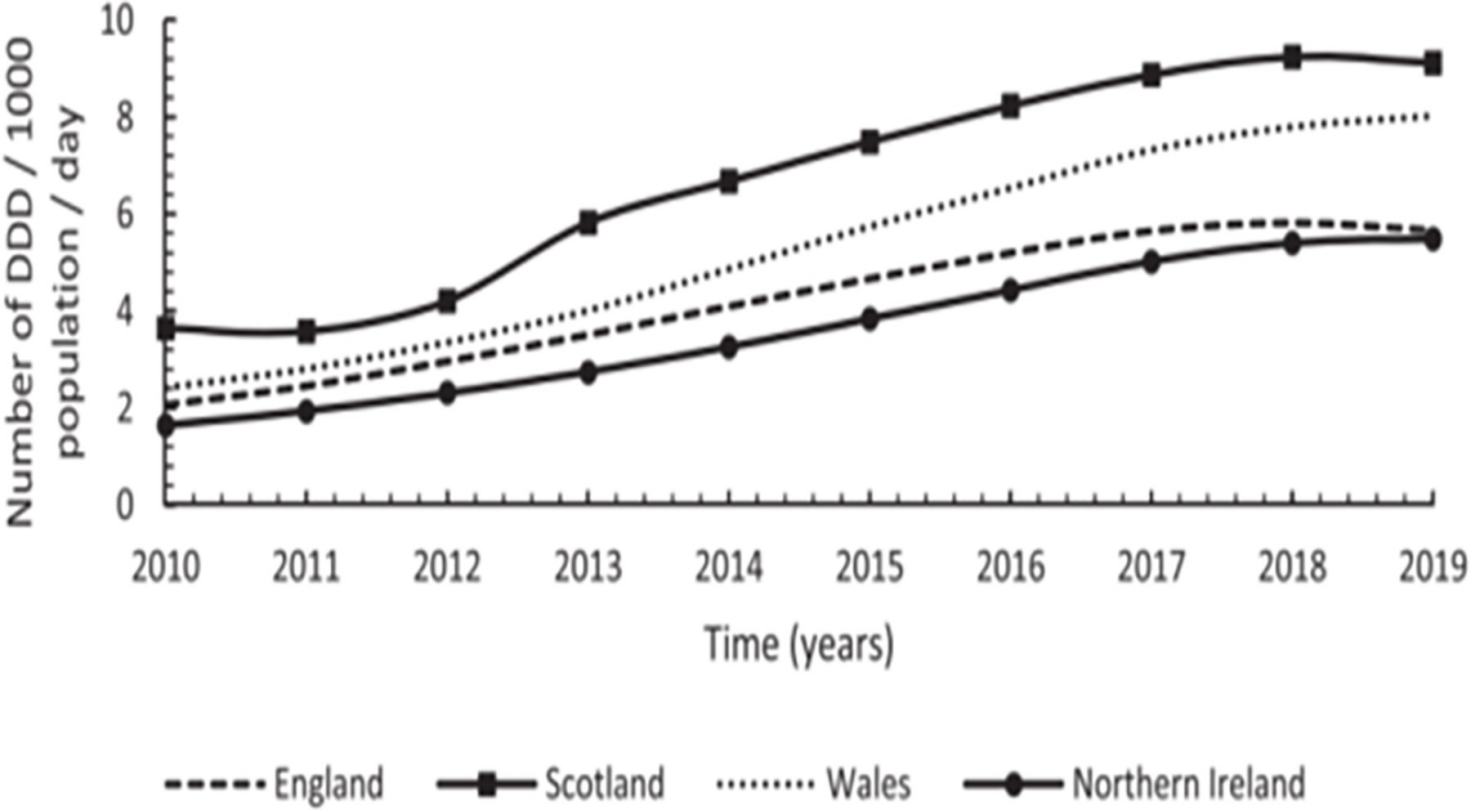
Annual utilization trend in number of DDD/1000 inhabitants/day for gabapentin across UK between 2010–2019. Source: Kurdi (2021) Licensed under CC BY 4.0.

Three studies investigated gabapentinoids prescribing patterns in Scotland. All employed observational designs [70, 75, 77]. Gabapentinoids prescribing patterns in Scotland between 2006–2016 increased quadruply from 164 650 in 2006 to 694 394 in 2016. Regional health boards also had significant gabapentin increase between 2006–2016, with NHS Tayside recording a 218.5% increase, and NHS Fife a 249.7% increase. Although pregabalin statistics were not specified, the authors concluded that it had similar increasing rates [77]. Data on gabapentinoids utilisation in Scotland showed that there was a yearly increase in gabapentinoids between 2010–2019, however after 2016 this trend slightly plateaued due to lower rates of gabapentin prescriptions (Fig 4) [70, 75]. Compared to other UK nations, Scotland had the highest utilisation level and annual increase in gabapentinoids rates between 2010–2019, with the highest utilisation trend for gabapentin, and the highest increase alongside Wales (Fig 4) [70].

**Fig 5:**
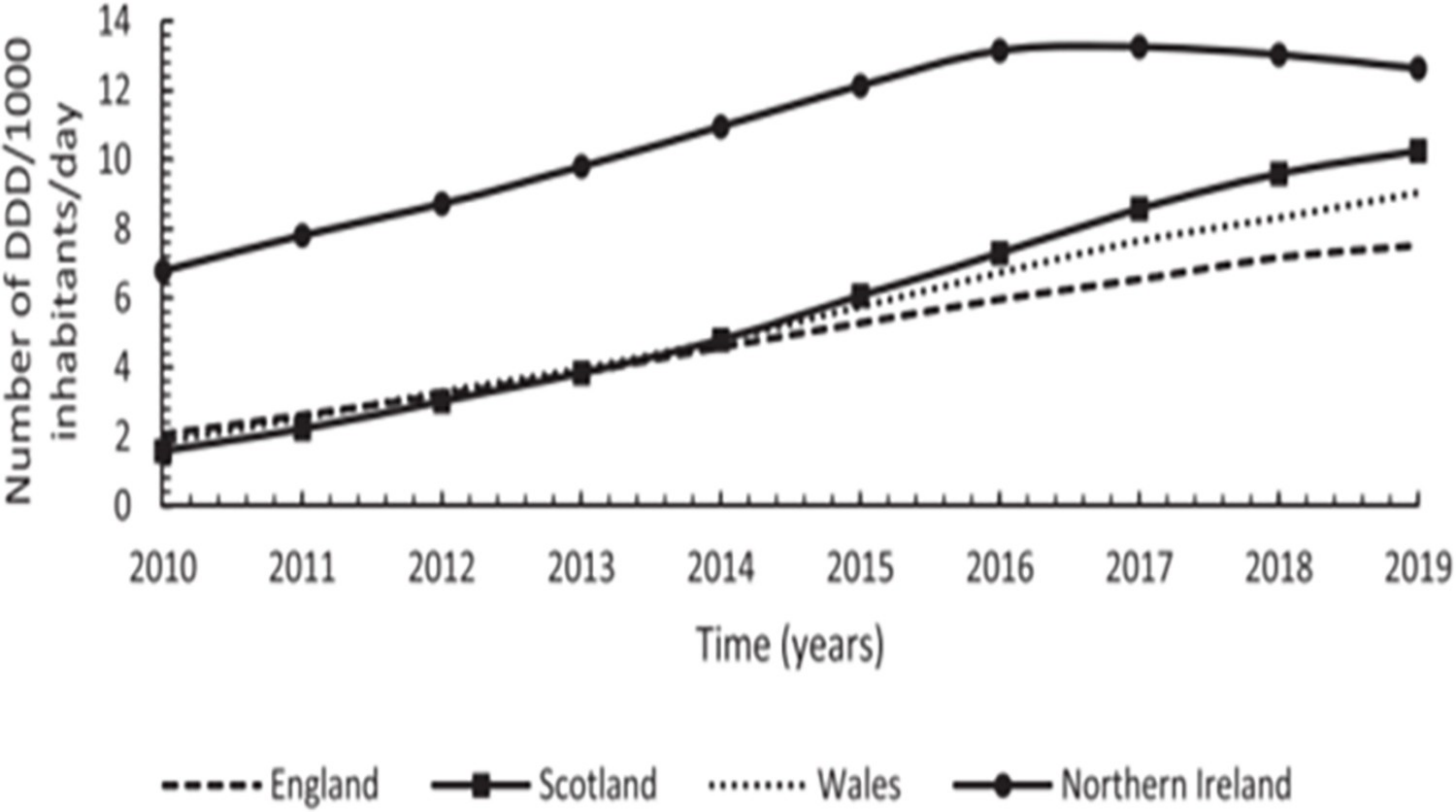
Annual utilization trend in number of DDD/1000 inhabitants/day for pregabalin across UK between 2010–2019. Source: Kurdi (2021) Licensed under CC BY 4.0.

Scotland has shown a trend of high use of pregabalin following Northen, with having the highest and consistent annual yearly increase overall (Fig 5) [70, 75].

#### 3.6.2. Fatal trends

Two observational studies [70, 77] and one systematic review on overdoses in Scotland and England [79] provided data on GRDs.

**Fig 6:**
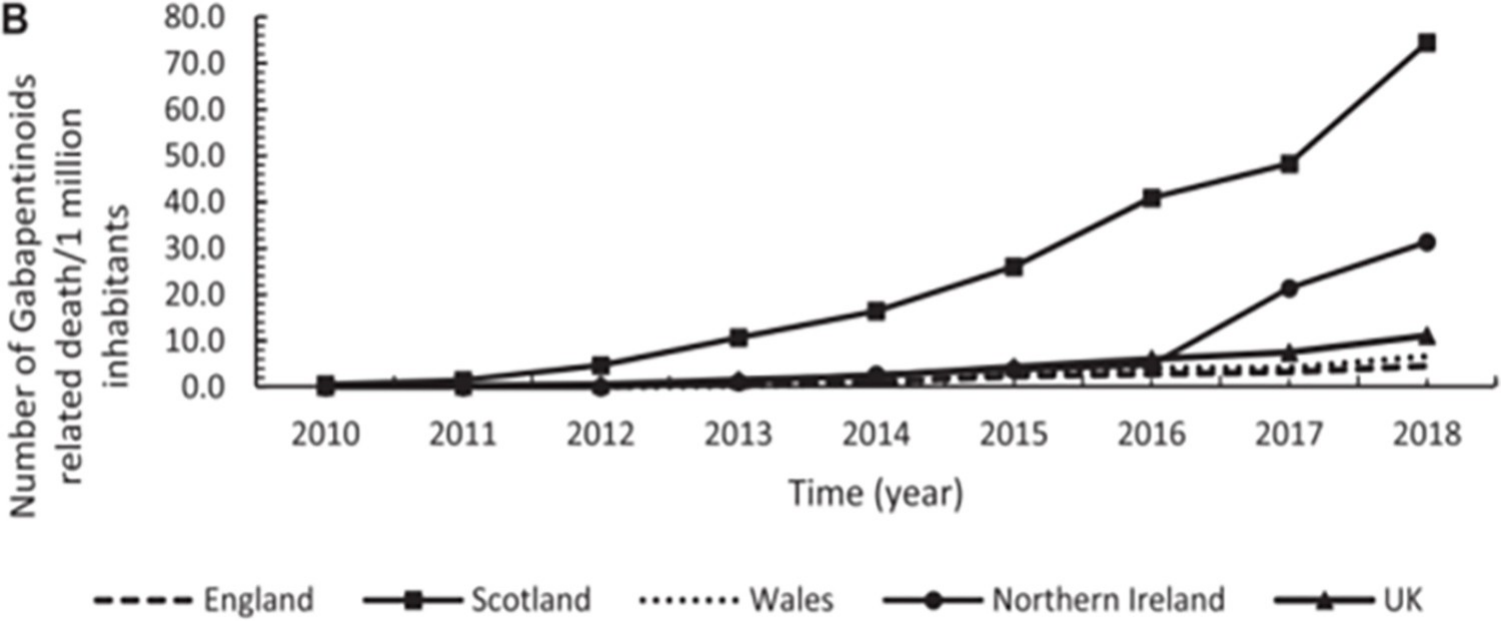
Annual trends in number of deaths per million inhabitants for gabapentinoids across the UK between 2010–2018. Source: Kurdi (2021) Licensed under CC BY 4.0.

Both observational investigations used data from National Records Scotland (NRS) NRS and Office for National Statistics (ONS) and observed high prevalence of GRDs [70, 77]. Scotland had a 12-fold increase in GRD between 2010–2018, and a significant yearly increase of 8.7 deaths per million inhabitants [70] exceeding other UK nations (Fig 6).

In 2017, NHS Tayside had higher GRD increase rate compared to Scotland, with increase of 23% gabapentin deaths compared to 14% in Scotland and increase of 33% pregabalin deaths compared to 12% in Scotland [77].

GRDs in Scotland surged since 2015 (Fig 6). By 2016, gabapentinoids were the most common substances in Scottish DRDs toxicology following opioids and benzodiazepines [77], and by 2018 they were implicated in 31% of all Scottish DRDs [79]. These findings indicate that gabapentinoids are still prevalent, irrespective of the slight plateau observed in their prescription rates [70, 75].

### 3.7. Summary of etizolam and gabapentinoids use prevalence

In summary, there is a high prevalence of etizolam and gabapentinoids in Scotland. Both etizolam and gabapentinoids related deaths have shown recent increase, with a substantial rise since 2015. Data on seized drugs adds to the narrative of etizolam prevalence showing high detection rates in Scottish prisons between 2020–2021. Data on gabapentinoids utilisation shows high prescription rates in Scotland highlighting the prevalence of these substances.

### 3.8. Determinants of adverse effects and fatality

#### 3.8.1. Etizolam

Polydrug use was the most common determinant of etizolam adverse effects in the identified literature. Out of 806 etizolam related deaths in Scotland in 2020 only 1% was caused solely by etizolam, with the remaining DRDs involving other substances [6]. Analysis of 162 Scottish DRDs due to drowning showing high number of cases of DBDZ related deaths between 2013–2022, with most deaths occurring between 2019–2020 and driven by DBZD including etizolam (46.9%) usually alongside other substances. Most common combination of drugs (17.5%) included DBZD and opioids [66]. An analysis of postmortem cases in Fife Scotland investigated underlying cardiovascular pathologies in DRDs between 2003–2019 [63]. Out of 436 cases, DBDZ (alprazolam, diazepam and etizolam) were found in 34.4% and were significant predictors for cardiovascular pathologies. Polydrug use was common with 75.8% cases involving opioids, 22% cannabinoids and 22% gabapentinoids. Although the study did not analyse the predictive value of polydrug use on cardiovascular pathology, they concluded that this effect cannot be ruled out given its high prevalence [63].

Out of 370 Scottish alprazolam related deaths due to accidental poisoning between 2013–2020, “other” benzodiazepines were involved in 67.21%, of which etizolam was implicated in 22.4%. Polydrug use was common with mean number of substances involved in deaths reaching five, with most common combination being DZBD and opioids [67]. Analysis of 19 ethylphenidate related deaths between 2013–2014 in Edinburgh found that two deaths involved etizolam alongside other substances, with 52.6% of all cases caused by multidrug toxicity [74].

Quantitation of DBZD in Scottish prisons provides evidence of etizolam polydrug use as well other determinants of its adverse effects [72]. Fig 3 shows combination of etizolam and different DBZD detected, with over 11% of samples including a mixture of etizolam and flubromazepam. Other than DBZD and etizolam mixtures, commonly a mixture of etizolam and synthetic cannabinoids was detected [72].

Unintentional ingestion of etizolam alongside or instead of other substances was a common finding. Analysis of detected etizolam tablets shown that many had similar markings as diazepam produced by pharmaceutical companies [72]. Out of these types of tablet detected, only 27.4% contained diazepam, with 45.3% containing solely etizolam. Considering, “unmarked blotters” (blotters which do not have any identifiable markings e.g. logos, numbers, symbols) which were first detected in Scottish Prisons in 2021 and grew in prevalence by 2022, they mostly contained etizolam or bromazolam (Fig 3), however they have also been detected to contain high doses of novel synthetic opioids. These were indistinguishable from those containing etizolam [72]. Analysis of seized illicit tablets by Police Scotland shown that etizolam tablet marking of “MA/D10” was found on many tables containing only diazepam or phenazepam [65].

**Fig 7:**
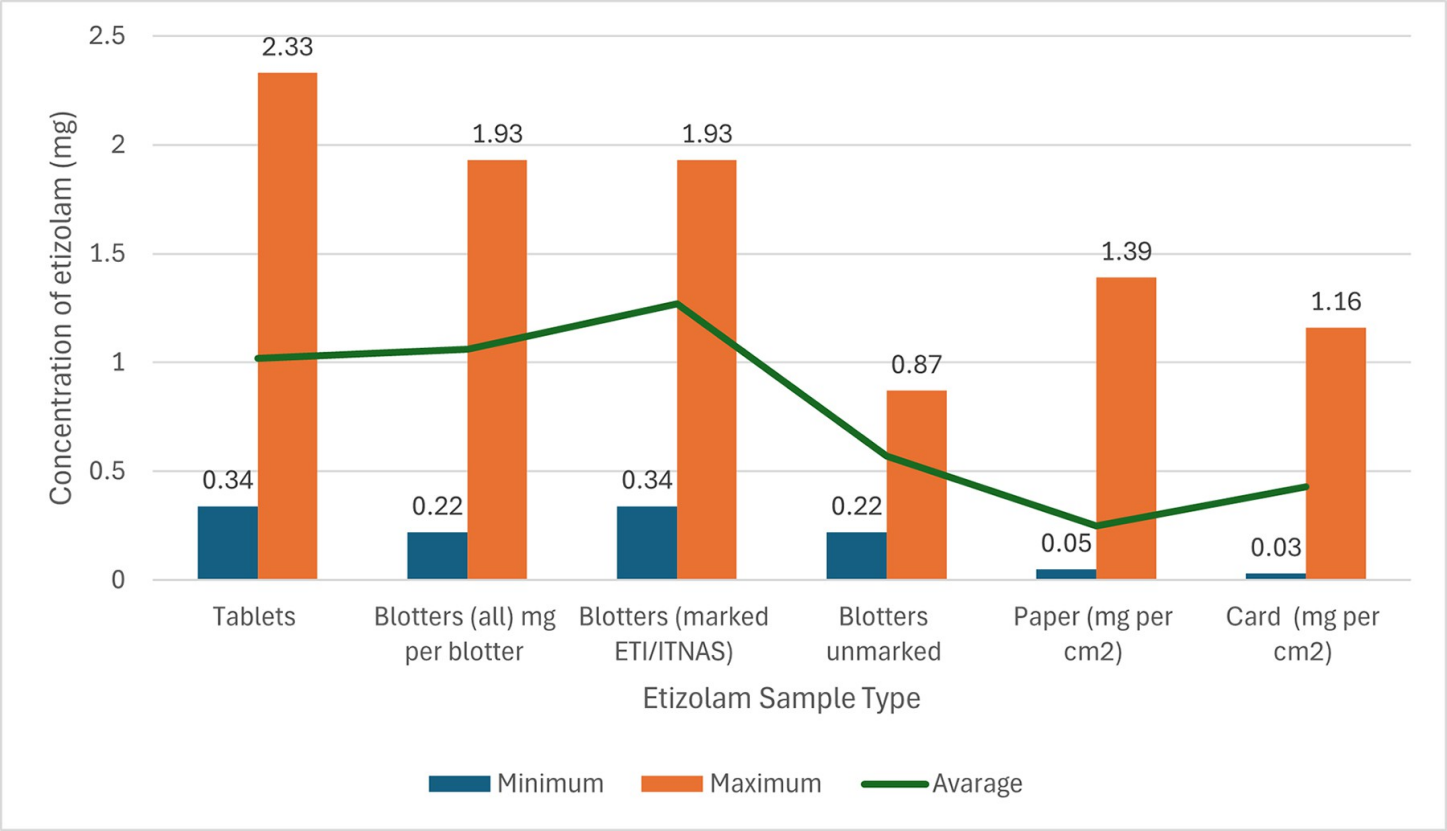
Quantitation of etizolam samples seized in Scottish Prisons between February 2019–2023 Source: Source: Adapted from Marland et al (2023) original table. Licensed under CC BY 4.0.

Inaccurate dosing of etizolam was also determined, with quantitation of etizolam content across one card seized in Scottish Prison shown a range from 1.16 to 1.87mg/cm2, with the entire card being equivalent to 306 tablets. Furthermore, quantitation of 193 etizolam samples using validated method (GC-MS) revealed high variability of etizolam within and across samples, ranging between 0.25 to 2.33mg (Fig 7) [72].

#### 3.8.2. Gabapentinoids

Polydrug use alongside gabapentinoids was a common finding with studies showing its contribution to the potential adverse effects [64, 67, 69, 74, 79].

Investigation of non-fatal overdose (NFOD) revealed that out of 439 identified NFOD in Tayside Scotland between 2017–2019, 61 cases were attributed to gabapentinoids (13.1%), however only one case was a sole gabapentinoids NFOD with 44 cases also involving methadone and 28 cases involving heroin [69]. This provides evidence that gabapentinoids can induce a NFOD, but this is more likely when combined with opioids. The adverse effects of gabapentinoids use alongside opioids is also illustrated by recent review findings. Gabapentinoids in combination with heroin/methadone was implicated in 23% of Scottish DRDs in 2016, and gabapentinoids were implicated in 36% of opioid overdoses in 2018 [79].

Baird et al [64] survey on individuals with SUD in Lothian, Scotland was the only identified study that investigated motives for gabapentinoids misuse, and the findings revealed that gabapentinoids users often co-use opioids. Out of the individuals misusing gabapentinoids, 36% self-reported using them to enhance the effect of methadone, e.g. “bring on the highest peak of methadone” [64].

The current review also identified two observational studies which showed that gabapentinoids use is common alongside other substances. An investigation into 370 alprazolam related deaths between 2004 to 2020 revealed that gabapentinoids were implicated in 42.9% of these cases with pregabalin found in 29.51% and gabapentin found in 18.3%, showing co-use of gabapentinoids and DBZD [67]. An investigation into 19 ethylphenidate related deaths revealed that two involved gabapentinoids with multidrug toxicity being the prominent cause for these deaths [74]. Due to design limitations, these studies did not identify whether the gabapentinoids use was prescribed or illicit.

The current review Identified that concurrent gabapentinoids prescribing Is prominent In Scotland. A study of new gabapentinoids patients [75] showed that recently the rate of concomitant opioid prescriptions has rose proportionally with the rise of new gabapentinoids patients in Scotland. Likewise, in Tayside and Fife areas co-prescribing was common with 60% of individuals receiving gabapentinoids prescriptions also receiving opioids or benzodiazepines or both [77]. Furthermore, out of 46 GP practices in Fife, 12.4% of gabapentinoids patients were concurrently prescribed opioids, with 9.36% prescribed strong doses [76].

**Fig 8:**
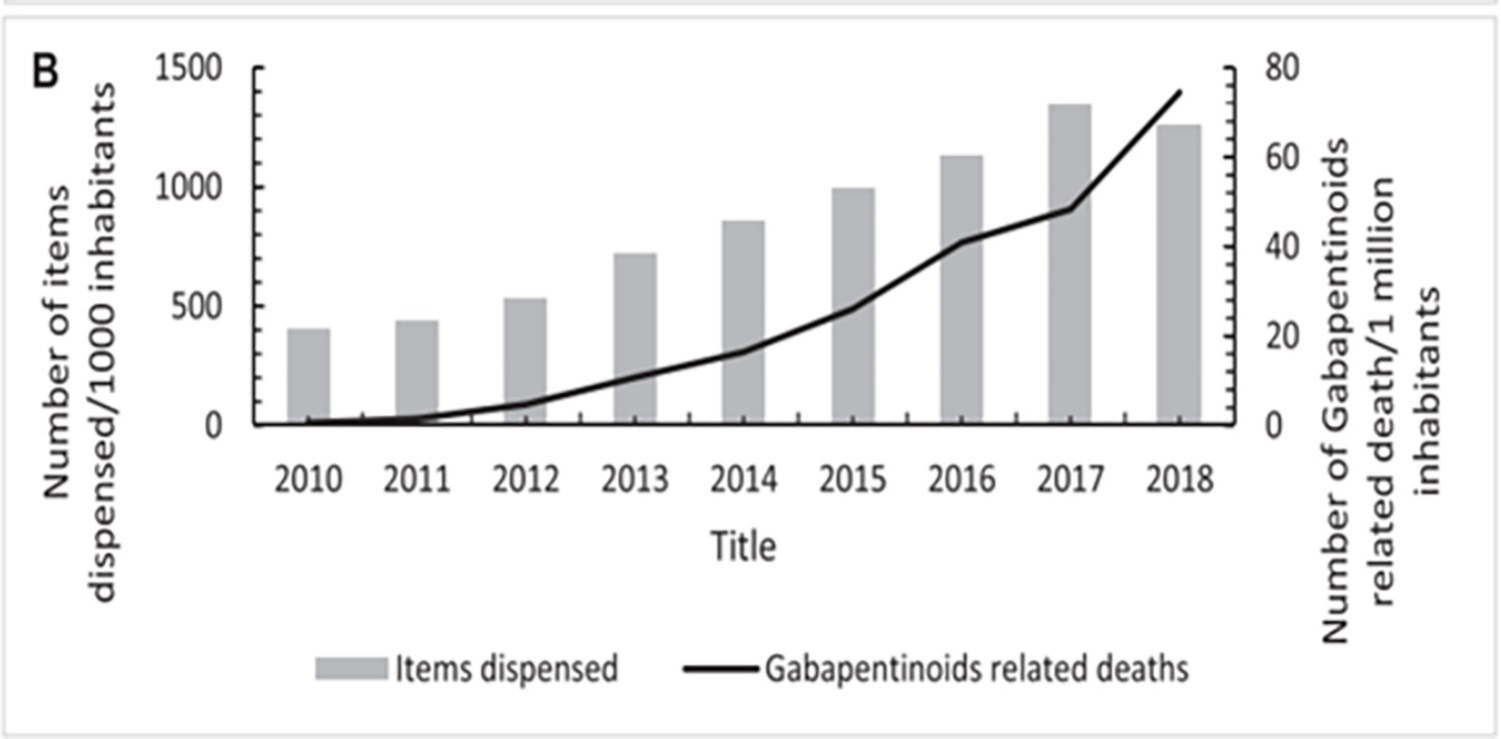
Annual trends in GRD in association with its utilisation trends across Scotland 2010–2018. Source: Source: Kurdi (2021) Licensed under CC BY 4.0.

These studies did not investigate the impact of the concurrent prescribing on gabapentinoids adverse effects. Data on gabapentinoids utilisation showed that their prescription increase in Scotland significantly correlated with the increase in GRDs (Fig 8) [70].

Furthermore, opioid prescription utilisation and related deaths had a similar trajectory of those involving gabapentinoids [70], showing possible correlation.

Notably, recent gabapentinoids utilisation slightly plateaued but this did not affect continuous increase in GRD (Fig 8) [70]. Torrence et al. [77] investigation showed that despite the high rates of gabapentinoids prescribing and concurrent opioids prescriptions, most GRD in Tayside Scotland involved non-prescribed use of gabapentinoids [77]. These findings indicate that high diversion rates and poly drug use could be impacting GRD.

#### 3.8.3. Summary

In summary, the current literature indicates that concurrent use of opioids and other substances is the most common determinant of both etizolam and gabapentinoid related adverse effects and fatality in Scotland. Gabapentinoids utilisation data also implicated concurrent prescribing and diversion in Scotland with its possible relation to GRDs. Etizolam quantitation data showed how illicitly manufactured etizolam supply in Scotland can cause higher risk of unintentional polydrug use and inaccurate dosing, further impacting on etizolam related mortality and adverse effects.

### 3.9. Characteristics of at-risk users

#### 3.9.1. Etizolam

Only one study provided information on several characteristics found in individuals using DZBD in Scotland including etizolam.

Corkey et al. [66] investigation of Scottish DRDs caused by drowning have characterised individual differences of the deceased. descendents. Given that etizolam alongside other DBZD was the main contributor to these deaths, the insights of this study could be important to the broader context of characteristics of individuals at risk of adverse effects from etizolam misuse [66]. Most of the descendents were found to be male (68.7%) and the mean age was above 38 years old. Notably, the male descendents in this study were found to be younger than their female counterparts (38.84 vs 43.64) and most common cause of death for males was found to be accidental, whereas female most common cause was intentional. This shows a possible vulnerability for etizolam adverse effects for older cohorts and possibly predominance of intentional misuse in females [66].

#### 3.9.2. Gabapentinoids

This review identified three studies which characterised individual differences in gabapentinoids users [77–79].

Torrence et al. [77] showed high prevalence of women being prescribed gabapentinoids in Scotland [77]. The investigation revealed that many gabapentinoids prescriptions issued were recurrent, with 73.32% patients issued three or more prescriptions. Regardless of the type of user females had the highest rates of prescription issued compared to males [77].

Average age of individuals prescribed gabapentinoids was 58, and most lived in deprived areas. The co-prescribing of opioids and benzodiazepines alongside gabapentinoids was most prominent among females, especially in older cohorts [77]. Illicit combined use of Methadone and gabapentinoids was found to be mostly prominent in older populations in Scotland [79].

**Fig 9:**
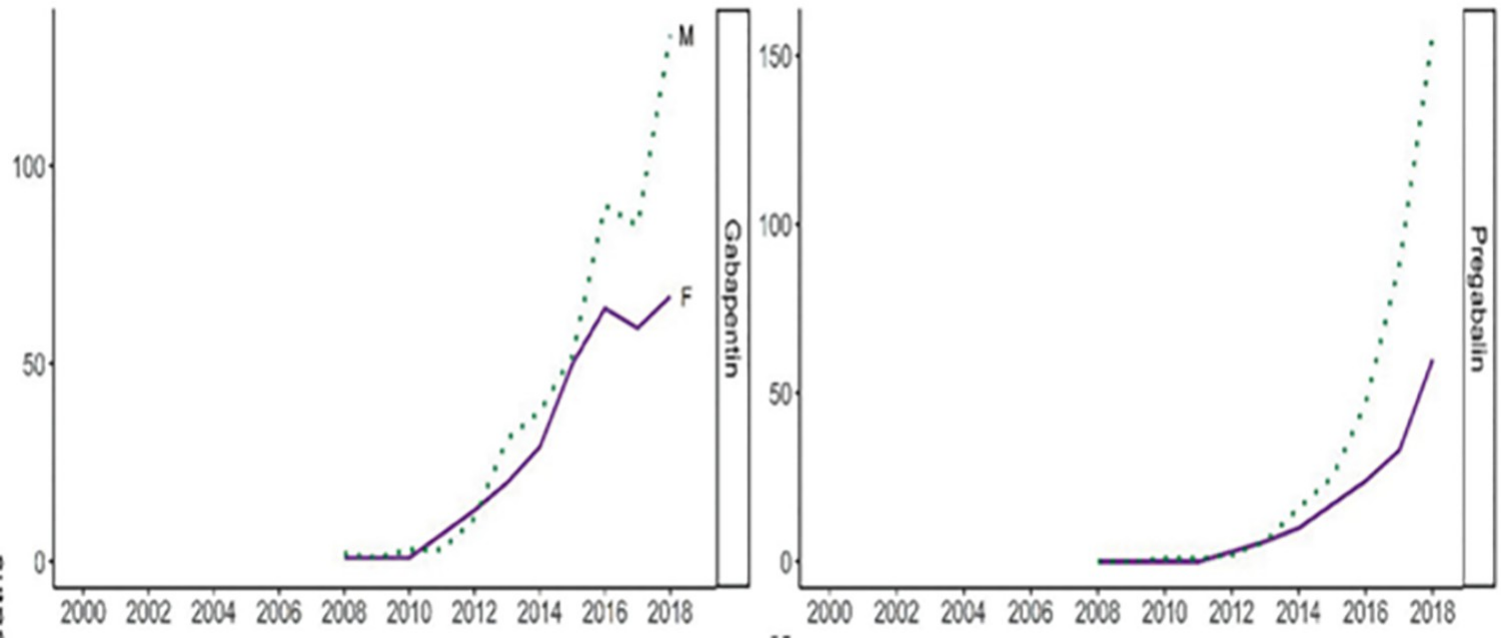
Number of GRD by gender: ONS ‘wide’ definition. Source: Tweed et al (2020) Licensed under CC BY 4.0.

Conversely, GRD data in Scotland do not corroborate these findings. In Tayside in 2016 most GRDs involved younger males. Notably, most of these deaths involved non prescribed use of gabapentinoids [77]. Recent mixed method analysis investigating the current increase of women DRDs in Scotland provides an account for this disparity [78]. Tweed et al. [78] reviewed DRDs statistics in Scotland, found that the substances reported in DRDs may differ depending on the used source and definition of DRDs. Tweed et al. [78] found that NRS statistics portray that in recent GRDs the trend over time was similar for both genders but much steeper for men. Conversely, The Office for National Statistics (ONS) data using “wide” definition for DRDs (ONS “wide” definition counts all drug poisoning deaths as DRD’s including prescribed drugs and substances not listed under the UK Misuse of Drugs Act 1971 [14] showed the difference in increase rate of GRD between genders was smaller (Fig 9).

**Fig 10:**
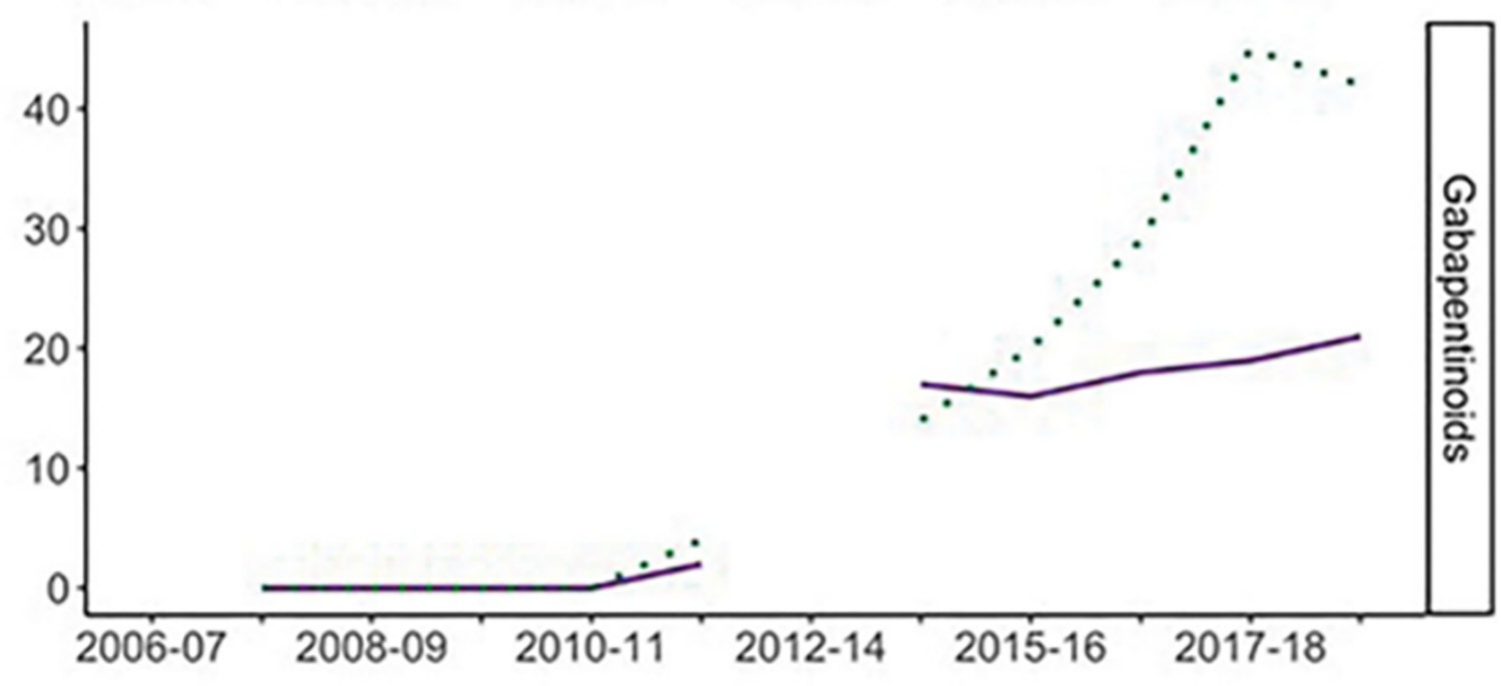
Number of individuals presenting for initial assessment in specialist drug services for gabapentinoids between 2006–2007 and 2017–2018 by gender. Source: Tweed et al (2020) Licensed under CC BY 4.0.

Interviews with stakeholders corroborate this, with findings that gabapentinoids prescriptions were of particular concern and are currently treated as a possible determinant for the increase in women DRDs in Scotland [78]. This vulnerability for women GRD is further exemplified by investigation of attendance for initial assessment in specialist drug services in Scotland showing lower attendance rates for women (Fig 10) [78].

#### 3.9.3. Summary

The Scottish literature for individual characteristics of at-risk users of gabapentinoids and etizolam is limited. Nevertheless, the identified studies indicate that both substances show predominance in older cohort. Gabapentinoids prescription utilisation and fatality data also indicates possible vulnerability in older females with gabapentinoids prescriptions.

## 4. Discussion

The increase in prescription drugs and their use as illicit drugs as well as the increase in average number of substances involved in Scottish DRDs has been established as a possible explanation for the rise in DRDs in Scotland [4, 5]. Whilst opioids, cocaine and alcohol have consistently potentiated the prevalence of polydrug use in Scotland, there is clearly a shift towards other drugs. The literature included in this review indicates that significantly DZBD such as etizolam, and gabapentinoids have increased in prevalence amongst PWUD in Scotland [6, 7]. Therefore, the present review aimed to summarise the Scottish literature on gabapentinoids and etizolam and establish their contribution to the rise in Scottish DRDs, focusing on their use prevalence, their adverse effects and its contributors, and individual characteristics of at-risk users.

### 4.1. Prevalence of gabapentinoids and etizolam

Despite this increase in use patterns the literature on gabapentinoids and etizolam misuse is limited. However, the findings retrieved from studies investigating DRDs trends in Scotland were consistent with the reported increase in the use of gabapentinoids and etizolam. There has been a substantial rise in fatalities in recent years involving both substances, with first spike in related deaths documented in 2015. This pattern has also been documented in England and US [10, 41]. In this review it is evidenced that Scotland is experiencing a significant rise in deaths involving gabapentinoids and etizolam [6, 70].

The increase in etizolam prevalence in Scotland is further exemplified by studies quantifying seized drugs [65, 72]. Scottish prisons showed high etizolam detection rates compared to other NPS between 2020–2021 [72]. Findings from a recent review on NPS prison detection rates has substantiated this, showing that most prevalent NPS in Scottish prisons until 2015 were synthetic cannabinoids, with etizolam detected thereafter [80]. Data on prescription use of gabapentinoids in Scotland has emphasized their prevalence, with a high yearly increase in prescriptions issued for pregabalin [70, 75] and high but recently plateauing utilisation of gabapentin [70, 75, 77]. This reflects similar prescribing patterns reported in US and the UK [36–38]. This review demonstrates that gabapentinoids consumption in Scotland exceed other UK nations, especially when considering gabapentin use and pregabalin yearly increase rates [70, 75]. High gabapentinoids use has been previously associated with the increase in GRD in England [48]. The limited literature establishing causality between gabapentinoids use and GRD means that addressing this relationship was beyond the scope of this review. One investigation has found that gabapentinoids use significantly correlated with their fatality increase [70]. Furthermore, most deaths were due to non-prescribed use of gabapentinoids [77]. As gabapentinoids are mostly obtained from healthcare providers, family and friends [43], these findings suggest that gabapentinoids are being diverted to the unregulated market in Scotland, which could be a contributing factor to the reported increase in fatalities.

Overall, this review has identified that gabapentinoids and etizolam prevalence in Scotland is increasing and is potentially contributing to the rise in Scottish DRDs. The current literature, however, is limited, lacking in methodological variety and quantity. Large registers can be effective at exemplifying trends over time, but they are not as effective at identifying individual factors and causal relationships [41]. Further evaluation of the misuse prevalence of etizolam and gabapentinoids is needed. This will provide important basis for clinical decision making, and enhance knowledge for drug regulators and authorities, aiding beneficial policies, and assessment of the changing drug use patterns in Scotland [81].

### 4.2. Adverse effects and polydrug use

Another point of concern is that the average number of substances found in DRDs in Scotland is increasing, indicating substantial polydrug use is contributing to gabapentinoid and etizolam fatalities as well as potential adverse effects. Most fatalities or overdoses where gabapentinoids or etizolam were implicated, were not due to their sole use [6, 69, 79]. Typically, other substances especially opioids, were reported to be present [66, 67, 79]. There are several possible explanations for the association between the use of these substances and opioids. Individuals were found to use etizolam or gabapentinoids alongside opioids to potentiate and enhance their euphoric effects, as well as aid withdrawal and self-medicate symptoms of anxiety or pain [10, 28, 50, 64, 82]. Furthermore, etizolam use alongside or instead of other substances can occur unintentionally [72, 65]. Given the substantial variation in sample concentrations and the finding that the drug obtained may not be what is advertised to be, with etizolam tablets often mimicking the look of other BDZD such as diazepam [65, 72], and etizolam blotters often containing synthetic opioids [72]. This is further supported by a recent review on global use of DBZD which found that etizolam fatalities usually occur due to accidental poisoning or multidrug toxicity [83]. Considering gabapentinoids, they are often co-prescribed alongside opioids and other substances [75–77]. Previous research shown that people who are prescribed gabapentinoids alongside opioids are more likely to misuse both substances and are often at higher risk for opioid related overdoses [45], indicating that concurrent prescribing can increase the chance of polydrug use and contribute to DRDs.

Given the contributory effect of polydrug use on gabapentinoids and etizolam adverse effects, there is a need for further research, especially in longitudinal population studies and basic research. This would allow for further exploration into the motives for co-using these substances and allow for explanations on the possible additive or synergetic effects of opioids, gabapentinoids and etizolam.

### 4.3. Characteristics of at-risk users

Establishing the unique characteristics of etizolam and gabapentinoids at-risk users is important for understanding factors contributing to their related fatalities and for guiding public health and regulatory measures to mitigate DRDs and protect vulnerable populations. This current review highlighted a lack of evidence indicating limited reporting in Scotland into characteristics of at-risk users of etizolam or gabapentinoids. For etizolam only one study provided substantial demographical analysis, showing that most at risk users were male and comprised of the ageing population (over 35 years old) [66]. This study also revealed that women users were older than their male counterparts and their deaths were of intentional nature. Interestingly, etizolam related deaths were found to occur mostly due to accidental poisoning [83], this illustrates a distinctive and unusual vulnerability in women who misuse etizolam. Notably, this study was focused solely on drug related drowning fatalities, which limits its generalisability.

The gabapentinoid related literature has also shown a distinctive vulnerability in older populations and women. Complex multidrug prescribing was found to be prevalent in older populations and women receiving gabapentinoids prescriptions [77]. Concurrent opioid misuse was also a common trend in older cohorts [79]. There was also an increasing fatality rate from prescribed gabapentinoids as well as poor treatment initiation being common in females [78]. These findings are corroborated by previous research in US and England where individuals receiving concurrent gabapentinoids and opioid treatment were found to be older and of female gender [84, 85], with a recent review showing that gabapentinoids detection has been consistently increasing in female toxicology reports [50].

Economic deprivation was also identified as a risk factor, with an association between high deprivation and concurrent drug prescribing [77]. A recent US based investigation revealed that stricter gabapentinoids prescribing regulations increased the risk of over prescribing and other gabapentinoids related harms in the most deprived states [81]. This is consistent with the current review, highlighting that Scottish GRD increased after the regulation in 2019, which classified gabapentinoids as a schedule three, class C drug [47, 70, 75]. Given this possible association, further research is needed into the efficacy of gabapentinoid regulations in Scotland, especially in highly deprived areas.

Overall, the current research investigating individual characteristics of gabapentinoids and etizolam in at risk populations is limited. However, the existing studies provide an important basis for future research, including investigations into the use of these substances in older populations, women, as well as the effects of deprivation.

### 4.4. Limitations of the studies

There are several methodological limitations in the reviewed studies. Much of the research on gabapentinoids and etizolam prevalence in DRDs was secondary observational analysis of large Scottish databases such as NRS or ONS. These databases are subject to limitation for example, limited scope for investigation of individual characteristics of using these drugs as well as their limited ability to establish any causality [41]. They are also subjected to bias and poor accuracy in determination of which drugs caused deaths. This is due in part to the constraints of Scottish DRDs toxicology reporting. Toxicology investigations tend to be limited with only an indication from the relevant pathologist as to which substances were implicated in or caused death with in-depth data on the level of drug in body tissue not provided [67]. Considering the variety of substances involved in a recorded death in relation to gabapentinoids or etizolam it is difficult to identifying which drug was responsible. This often causes the death to receive a general label of “multidrug toxicity” or the death is counted in more than one substance category [41]. This limits accurate reporting of gabapentinoids and etizolam related fatalities. Furthermore, toxicology testing for gabapentinoids and etizolam are also subjected to voluntary request, and often depended on witness reports, with full toxicology screening not conducted for all DRDs in Scotland, especially in deaths where opioid use was established and where further toxicology is not deemed necessary [48]. Recently, due to the increased awareness of gabapentinoids and etizolam adverse effects, and the regulatory changes toxicology testing requests have increased [6, 86]. This could in part explain their recent increase in detection shown by the studies in this review. Nevertheless, given the limitations, the current and past fatality trends of gabapentinoids and etizolam presented by the reviewed literature are almost certainly underestimated.

The studies included in this review have displayed methodological constraints, however these were mainly due to the limitations of Scottish DRD reporting and poor vigilance in toxicology testing. This has significant implications for guiding policy. Further investigation is warranted to understand the impact of these constrains on Scottish research investigating gabapentinoids and etizolam. This will merit future research improvements and aid accurate reporting of gabapentinoids and etizolam related fatalities in Scotland.

### 4.5. Limitations of the review

The current review has several limitations, firstly one paper which was omitted from the quality assessment [78], and one paper which was at high risk of bias [64] were included in the study. This could lead to an underestimation of the risk of bias in conclusions drawn from those studies. The decision to include those studies was however because it was deemed important to include all published research in respect of gabapentinoids and etizolam use in Scotland to provide most current information on the adverse effects of these drugs and their impact on Scottish DRDs. Nevertheless, to mitigate the potential impact of this limitation, rigorous study selection following PRISMA guidelines with inclusion and exclusion criteria was applied, as well as commentary on the methods for each study provided, and qualitative evaluations of their most prominent limitations conducted. This ensured comprehensive evaluation of the overall quality and reliability of synthesised evidence. The current review also did not include non-peer reviewed studies in the synthesis, which could have imposed publication bias, however, peer reviewed studies add credibility, accuracy and validity to the evidence discussed in the review.

As we only included English language peer reviewed literature and restricted the search to PubMed and Google Scholar this could be considered a limitation. However, according to Bramer et al. [87] the use of these two search engines accounts for 93% of published literature on a given search.

### 4.6. Overall evaluations and policy and practice implications

Despite the limitations of the literature and the constraints of the review, the findings highlight the need for further research on the prevalence and risk of etizolam and gabapentinoids use in Scotland. Given the rise in Scottish DRDs and the public and individual harm this is causing, any measures that curb these fatalities are merited. Although etizolam and gabapentinoids differ in the way they affect Scottish drug markets with etizolam often manufactured domestically and gabapentinoids often prescribed and partly diverted into the black market, both substances were found to similarly drive the current culture of polydrug use in Scotland and contribute to the increasing pattern of Scottish DRDs.

The current review also highlights areas of focus for future prevention, intervention, and policy strategies. Firstly, education at individual and public health level needs to be improved. Individuals need to be made aware of the fact that fatalities from etizolam and gabapentinoids occur and that they are often accidental due to multidrug toxicity. Improved awareness of the dangers of polydrug use could mitigate harms and educate individuals that despite the notion that these substances are safe, they can yield severe harm. To promote better understanding of the prevalence of polydrug use, it would also appear prudent to include gabapentinoids and etizolam in standard toxicology screening. Considering etizolam, promoting awareness of the dangers of counterfeit tablets needs to be the next crucial preventive measure of its harms, especially in Scottish prisons. The current review also shown the possible correlation of gabapentinoids overprescribing and the rise of GRD. More guidance should be given to GP’s regarding prescribing of these substances with careful examination into frequency and variety of the drugs that are given, especially to individuals with SUD.

Lastly the current regulatory measures for etizolam and gabapentinoids have not been successful so far in curbing fatalities and drug related harms This is especially the case in Scotland, where their use, and related harm is still highly prevalent. Therefore, the existing systems and polices in Scotland require improvements through expanding current research on these substances. Higher quality research, with a wider range of methodologies would help inform policy. It is also important to include addiction treatment providers, prescribers and individuals who use these substances in future research. Taking this approach could help shape positive change in the current systems, interventions, and surrounding policies, in turn mitigating the current rise in DRDs in Scotland.

## 5. Glossary of terms

**Polypharmacy–**The practice of prescribing or taking multiple medications concurrently to manage a particular condition or multiple conditions, which could lead to potential drug interaction and risk of adverse effects.

**Polydrug use**–The concurrent use of more than one drug or type of drug simultaneously or sequentially, whether for recreational, therapeutic or self-medication purposes, typically associated with recreational use and leading to complex dependence and addiction patterns.

**Adverse event**–Any incident in which a drug causes and/or contributes to negative outcomes including side effects, complications, or unintended reactions.

**Determinant of adverse effect**–The range of biological, environmental, social and behavioural factors that influence any incidence and/or severity of adverse effects. This could include overall drug intake, frequency of use, risky behaviours, polydrug use etc.

**Drug Toxicity**–A negative and harmful effect on the body caused by a drug, or multiple drugs (multidrug toxicity) resulting from either excessive dosage, long-term use, or polydrug use.

## Supporting information

S1 Table Table 1. Prisma checklist and Table 2. JBI appraisal. (DOCX)

S2 Table Table of studies identified in the initial search. (XLSX)

S3 Table Table of data extraction. (XLSX)

## List of abbreviations

DRD: Drug related deaths
DZBD: Designer benzodiazepine
GRD: Gabapentinoid related deaths
PWUD: People who use drugs
NPS: Novel psychoactive substances
SUD: Substance use disorder
